# A Schwarz Lemma for the Pentablock

**DOI:** 10.1007/s12220-022-01107-7

**Published:** 2022-12-19

**Authors:** Nujood M. Alshehri, Zinaida A. Lykova

**Affiliations:** School of Mathematics, Statistics and Physics, Newcastle University, Newcastle upon Tyne, NE1 7RU UK

**Keywords:** Inner functions, Pentablock, Schwarz lemma, Distinguished boundary, Primary 32F45, 30E05, 93B36, 93B50

## Abstract

In this paper, we prove a Schwarz lemma for the pentablock. The pentablock $\mathcal {P}$ is defined by

$$\begin{aligned} \mathcal {P}=\{(a_{21}, {\text {tr}}A, \det A) : A=[a_{ij}]_{i,j=1}^2 \in \mathbb {B}^{2\times 2}\} \end{aligned}$$

where $\mathbb {B}^{2\times 2}$ denotes the open unit ball in the space of $2\times 2$ complex matrices. The pentablock is a bounded non-convex domain in $\mathbb {C}^3$ which arises naturally in connection with a certain problem of $\mu$-synthesis. We develop a concrete structure theory for the rational maps from the unit disc $\mathbb {D}$ to the closed pentablock $\overline{\mathcal {P}}$ that map the unit circle $\mathbb {T}$ to the distinguished boundary $b\overline{\mathcal {P}}$ of $\overline{\mathcal {P}}$. Such maps are called rational ${\overline{\mathcal {P}}}$-inner functions. We give relations between ${\overline{\mathcal {P}}}$-inner functions and inner functions from $\mathbb {D}$ to the symmetrized bidisc. We describe the construction of rational ${\overline{\mathcal {P}}}$-inner functions $x = (a, s, p) : \mathbb {D} \rightarrow \overline{\mathcal {P}}$ of prescribed degree from the zeroes of *a*, *s* and $s^2-4p$. The proof of this theorem is constructive: it gives an algorithm for the construction of a family of such functions *x* subject to the computation of Fejér–Riesz factorizations of certain non-negative trigonometric functions on the circle. We use properties and the construction of rational ${\overline{\mathcal {P}}}$-inner functions to prove a Schwarz lemma for the pentablock.

## Introduction

An unsolved problem in $H^\infty$ control theory led us to consider inner rational mappings from $\mathbb {D}$ to certain domains in $\mathbb {C}^d$ which arise in connection with the $\mu$-synthesis problem. One such domain is the pentablock. Other well-known examples of such domains are the symmetrized bidisc $\Gamma$ and the tetrablock. We should mention papers on the construction of rational $\Gamma$-inner functions [5, 6] and rational tetra-inner functions [15, 16] for the symmetrized bidisc $\Gamma$ and the tetrablock, respectively. The pentablock $\mathcal {P}$ was introduced by Agler, Lykova and Young in [4] in 2015. It was shown there that $\mathcal {P}$ arises naturally in the context of $\mu$-synthesis.

### Definition 1.1

[4] The open pentablock is the domain defined by

1.1 $$\begin{aligned} \mathcal {P}=\{(a_{21}, {\text {tr}}A, \det A) : A=[a_{ij}]_{i,j=1}^2 \in \mathbb {B}^{2\times 2}\}, \end{aligned}$$

where $\mathbb {B}^{2\times 2}$ denotes the open unit ball in the space of $\;\;2\times 2$ complex matrices with respect to the operator norm arising from the standard inner product on $\mathbb {C}^2$.

Recall [20] that the *structured singular value* $\mu _E$ of $A\in \mathbb {C}^{m\times n}$ corresponding to subspace *E* of $\mathbb {C}^{n\times m}$ is defined by

1.2 $$\begin{aligned} \frac{1}{\mu _E(A)} = \inf \{\Vert X\Vert : X\in E \text{ and } \det (1-AX)=0\}. \end{aligned}$$

The cost function $\mu _E$ plays a central role in the “$H^\infty$ approach” to the problem of stabilizing a linear system in a way that is maximally robust with respect to structured uncertainty. This approach, developed and promoted by Doyle and Stein [21], reduces the “robust stabilization problem” to the solution of a variant of the classical Nevanlinna–Pick problem for matrix-valued functions, in which the cost function to be minimized is given by $\mu _E$ for some uncertainty space *E*, in place of the usual operator norm.

To date there is not a satisfactory mathematical treatment of this “$\mu$-synthesis problem” in general, and so mathematicians have studied some special cases, such as for $2\times 2$-matrix-valued functions and for some natural choices of the space *E*. In particular the authors of [4] investigated the following special case of $\mu _E$.

### Definition 1.2

Let

$$\begin{aligned} E=\mathrm {span~}\left\{ 1,\begin{bmatrix} 0&{}1\\ 0&{}0 \end{bmatrix}\right\} \subset \mathbb {C}^{2\times 2}, \end{aligned}$$

$\mathcal {P}_\mu$ is the domain in $\mathbb {C}^3$ given by

1.3 $$\begin{aligned} \mathcal {P}_\mu = \{(a_{21},{\text {tr}}A,\det A): A\in \mathbb {C}^{2\times 2}, \, \mu _E(A)<1\} \subset \mathbb {C}^3. \end{aligned}$$

It was proved in [4] that $\mathcal {P}=\mathcal {P}_\mu$.

The pentablock $\mathcal {P}$ is a region in 3-dimensional complex space which intersects $\mathbb {R}^3$ in a convex body bounded by five faces, comprising two triangles, an ellipse and two curved surfaces [4]. The closure of $\mathcal {P}$ is denoted by $\overline{\mathcal {P}}$.

In this paper, we study rational $\overline{\mathcal {P}}$-inner functions. We define a rational $\overline{\mathcal {P}}$-inner function to be a rational analytic function from $\mathbb {D}$ into $\overline{\mathcal {P}}$ which maps $\mathbb {T}$ into $b\overline{\mathcal {P}}$, where $b\overline{\mathcal {P}}$ is the distinguished boundary of $\mathcal {P}$. The distinguished boundary $b\overline{\mathcal {P}}$ of $\mathcal {P}$ is

$$\begin{aligned} b\overline{\mathcal {P}} = \bigg \{(a, s, p) \in \mathbb {C}^3 : |s| \le 2, \ |p|=1, \ s = \overline{s}p \; \text {and} \; |a| = \sqrt{1-\frac{1}{4}|s|^2}\bigg \}, \end{aligned}$$

see [4]. The degree of a rational $\overline{\mathcal {P}}$-inner function $x = (a, s, p)$ is defined to be the pair of numbers $(deg \; a, deg \;p)$. We say that deg $x \le (m, n)$ if deg $a \le m$ and deg $p \le n$. The group of automorphisms of the pentablock was studied in [4] and [26].

Recall that a classical *rational inner function* is a rational map *f* from the unit disc $\mathbb {D}$ to its closure ${\overline{\mathbb {D}}}$ with the property that *f* maps the unit circle $\mathbb {T}$ into itself. A survey of results connecting inner functions and operator theory is given in [17]. All rational inner functions from the unit disc $\mathbb {D}$ to its closure ${\overline{\mathbb {D}}}$ are finite Blaschke products.

### Definition 1.3

[9, p. 2] A finite Blaschke product is a function of the form

1.4 $$\begin{aligned} B(z) = c\prod ^n_{i=1}B_{\alpha _i}(z) \ \ \ \ \text {for} \ z\in \mathbb {C}\setminus \{1/\overline{\alpha _1},\dots ,1/\overline{\alpha _n}\}, \end{aligned}$$

where $$B_{\alpha _i}(z) = \frac{z-\alpha _i}{1-\overline{\alpha _i}z}$$, $$|c|=1$$ and $$\alpha _1, \dots , \alpha _n \in \mathbb {D}$$.

We have proved several results on the description and the construction of rational $\overline{\mathcal {P}}$-inner functions and on the connections between rational $\Gamma$-inner functions and rational $\overline{\mathcal {P}}$-inner functions.

One of our main results is the construction of a rational $\overline{\mathcal {P}}$-inner function $x = (a, s, p)$ of prescribed degree from the zeros of *a* and *s* and $s^2 -4p$. The zeros of $s^2 -4p$ in $\overline{\mathbb {D}}$ are called the royal nodes of (*s*, *p*). One can consider this result as an analogue of the expression (1.4) for a finite Blaschke product in terms of its zeros. Concretely, the following result is a corollary of Theorem 8.6.

### Theorem 1.4

Let *n*, *m* be positive integers and suppose the following points are given

1. $$\alpha _1, \alpha _2, \dots , \alpha _{k_0} \in \mathbb {D}$$ and $$\eta _1, \eta _2, \dots , \eta _{k_1} \in \mathbb {T}$$, where $$2k_0+k_1=n$$;
2. $$\beta _1, \beta _2, \dots , \beta _m \in \mathbb {D}$$;
3. $\sigma _1, \dots , \sigma _n$ in $\overline{\mathbb {D}}$ which are distinct from $\eta _1, \dots , \eta _{k_1}$.

Then there exists a rational $\overline{\mathcal {P}}$-inner function $x = (a, s, p)$ of degree $\le (m+n, n)$ such that the zeros of *a* in $\mathbb {D}$ are $\beta _1, \beta _2, \dots , \beta _m$, the zeros of *s* in $\overline{\mathbb {D}}$ are $\alpha _1, \alpha _2, \dots , \alpha _{k_0}$, $\eta _1, \eta _2, \dots , \eta _{k_1}$, and the royal nodes of (*s*, *p*) are $\sigma _1, \dots , \sigma _n$.

There is a well-developed theory of Schwarz lemmas for various domains by many authors, including Dineen and Harris [19, 23]. In particular, for the symmetrized bidisc and the tetrablock, see [1, 11, 22]. Connections established in this paper between ${\overline{\mathcal {P}}}$-inner functions and $\Gamma$-inner functions (especially Theorem 7.8) and a Schwarz lemma for the symmetrized bidisc due to Agler and Young [11] allow us to prove a Schwarz lemma for the pentablock (Theorem 11.3).

### Theorem 1.5

Let $\lambda _0 \in \mathbb {D}\setminus \{0\}$ and $(a_0, s_0, p_0) \in \overline{\mathcal {P}}$. Then the following conditions are equivalent:

(i) there exists a rational $\overline{\mathcal {P}}$-inner function $x=(a, s, p)$, $x : \mathbb {D} \rightarrow \overline{\mathcal {P}}$ such that $x(0) = (0, 0, 0)$ and $x(\lambda _0) = (a_0, s_0, p_0)$;
(ii) there exists an analytic function $x=(a, s, p)$, $x : \mathbb {D} \rightarrow \overline{\mathcal {P}}$ such that $x(0) = (0, 0, 0)$ and $x(\lambda _0) = (a_0, s_0, p_0)$, and $|a_0| \; \le \; |\lambda _0| \sqrt{1-\frac{1}{4}|s_0|^2}$;
(iii) 1.5 $$\begin{aligned} \dfrac{2|s_0 - p_0\overline{s}_0| + |s_{0}^{2} - 4p_0|}{4 - |s_0|^2} \le |\lambda _0|\;\; \text {and} \;\;|s_0| <2, \end{aligned}$$
  and
  1.6 $$\begin{aligned} |a_0| \; \le \; |\lambda _0| \sqrt{1-\frac{1}{4}|s_0|^2}. \end{aligned}$$

The construction of an interpolating function $x=(a, s, p)$, $x : \mathbb {D} \rightarrow \overline{\mathcal {P}}$ such that $x(0) = (0, 0, 0)$ and $x(\lambda _0) = (a_0, s_0, p_0)$ is given in Theorems 11.2 and 11.3.

The authors are grateful to Nicholas Young for some helpful suggestions.

## The Pentablock $\mathcal {P}$ and the Symmetrized Bidisc $\Gamma$

In 1999, Agler and Young introduced the symmetrized bidisc in [10]. Following [10], we shall often use the co-ordinates (*s*, *p*) for points in the symmetrized bidisc $\mathbb {G}$, chosen to suggest ‘sum’ and ‘product’.

### Definition 2.1

The symmetrized bidisc is the set

2.1 $$\begin{aligned} \mathbb {G} {\mathop {=}\limits ^{\textrm{def}}} \{(z+w,zw):|z| \;<\; 1, |w| \; <\; 1\}, \end{aligned}$$

and its closure is

$$\begin{aligned} \Gamma {\mathop {=}\limits ^{\textrm{def}}} \{(z+w,zw):|z| \le 1, |w| \le 1\}. \end{aligned}$$

The following results from [2] give useful criteria for membership of $\mathbb {G}$, of the distinguished boundary $b\Gamma$ of $\Gamma$ and of the topological boundary $\partial \Gamma$ of $\Gamma$.

### Proposition 2.2

[2, Proposition 3.2] Let (*s*, *p*) belong to $\mathbb {C}^2$. Then

1. (*s*, *p*) belongs to $\mathbb {G}$ if and only if
  $$\begin{aligned} |s- \overline{s}p| \; <\; 1-|p|^2; \end{aligned}$$
2. (*s*, *p*) belongs to $\Gamma$ if and only if
  $$\begin{aligned} |s| \le 2 \textit{ and } |s- \overline{s}p| \le 1-|p|^2; \end{aligned}$$
3. (*s*, *p*) lies in $b\Gamma$ if and only if
  $$\begin{aligned} |p|=1, |s| \le 2 \textit{ and } s- \overline{s}p = 0. \end{aligned}$$

The following terminology was introduced in [12].

### Definition 2.3

The royal variety $\mathcal {R}_{\Gamma }$ of the symmetrized bidisc is

$$\begin{aligned} \mathcal {R}_{\Gamma } = \{(s, p) \in \mathbb {C}^2 : s^2 = 4p\}. \end{aligned}$$

### Lemma 2.4

[14, Lemma 4.3] Every automorphism of $\mathbb {G}$ maps the royal variety $\mathcal {R}_{\Gamma } \cap \mathbb {G}$ onto itself.

The royal variety is the only complex geodesic in the symmetrized bidisc that is invariant under all automorphisms of $\mathbb {G}$ [8].

### Remark 2.5

The pentablock is closely related to the symmetrized bidisc. Indeed, Definition 1.1 shows that $\mathcal {P}$ is fibred over $\mathbb {G}$ by the map $(a, s, p) \mapsto (s, p)$, since if $A \in \mathbb {B}^{2\times 2}$ then the eigenvalues of *A* lie in $\mathbb {D}$ and so $({\text {tr}}A, \det A) \in \mathbb {G}$. Thus, for every point $(a, s, p) \in \mathcal {P}$, the point $(s,p) \in \mathbb {G}$.

### Remark 2.6

In [26] Kosinski commented that the pentablock is a Hartogs Domain. It follows from the descriptions of the pentablock $\mathcal {P}$ in [4] that $\mathcal {P}$ can be seen as a Hartogs domain in $\mathbb {C}^3$ over the symmetrized bidisc $\mathbb {G}$, that is,

$$\begin{aligned} \mathcal {P} = \Big \{(a, s, p) \in \mathbb {D} \times \mathbb {G} : |a|^2 \; <\; e^{-\varphi (s, p)}\Big \}, \end{aligned}$$

where

$$\begin{aligned} \varphi (s, p) = -2\log \left| 1-\frac{\frac{1}{2}s{\overline{\beta }}}{1+\sqrt{1-|\beta |^2}}\right| , \end{aligned}$$

$$(s, p) \in \mathbb {G}$$ and $$\beta = \frac{s-\overline{s}p}{1-|p|^2}$$.

Hartogs domains are important objects in several complex variables.

### Definition 2.7

[24, p. 259] A domain $D \subset \mathbb {C}^n$ is called $\mathbb {C}$-convex if for any complex line $\ell = a+b\mathbb {C}, \; 0 \ne a, b \in \mathbb {C}^n$ such that $\ell \cap D \ne \emptyset$, this intersection $\ell \cap D$ is connected and simply connected.

It is known that the pentablock is polynomially convex and starlike, see [4]. It was shown in [31] that the pentablock $\mathcal {P}$ is hyperconvex and that $\mathcal {P}$ cannot be exhausted by domains biholomorphic to convex ones. Later in [30, Theorem 1.1] it was proved that $\mathcal {P}$ is a $\mathbb {C}$-convex domain.

The following results from [4] give useful criteria for membership of $\mathcal {P}$.

### Definition 2.8

[4, Definition 4.1] For $z \in \mathbb {D}$ and $(a, s, p) \in \mathbb {C}^3$ define $\Psi _z(a, s, p)$ by

2.2 $$\begin{aligned} \Psi _z(a, s, p) = \frac{a(1-|z|^2)}{1-sz+pz^2} \ \ \text { whenever } \ 1-sz+pz^2 \ne 0. \end{aligned}$$

The polynomial map implicit in the definition (1.1) can be written as

2.3 $$\begin{aligned} \pi (A)=(a_{21}, {\text {tr}}A, \det A) \; \text {for} \; A=[a_{ij}]_{i,j=1}^2 \in \mathbb {C}^{2\times 2}. \end{aligned}$$

Thus $$\mathcal {P} = \pi (\mathbb {B}^{2\times 2})$$.

### Theorem 2.9

[4, Theorem 1.1] Let

$$\begin{aligned} (s,p)=(\lambda _1+\lambda _2,\lambda _1\lambda _2) \end{aligned}$$

here $\lambda _1$, $\lambda _2$ $\in$ $\mathbb {D}$. Let a $\in \mathbb {C}$ and let

$$\begin{aligned} \beta =\frac{s-\overline{s}p}{1-|p|^2}. \end{aligned}$$

Then $|\beta | \; <\; 1$ and the following statements are equivalent:

1. (a,s,p) $\in \mathcal {P}$, that is, there exists $A \in \mathbb {C}^{2\times 2}$ such that $\Vert A\Vert < 1$ and $\pi (A) = (a, s, p)$;
2. $$|a| \; <\; |1-\frac{\frac{1}{2}s {\overline{\beta }}}{1+\sqrt{1-|\beta |^2}}|$$;
3. $$|a| \; <\; \frac{1}{2}|1-\overline{\lambda _2}\lambda _1|+\frac{1}{2}(1-|\lambda _1|^2)^\frac{1}{2}(1-|\lambda _2|^2)^\frac{1}{2}$$;
4. $$\sup _{z\in \mathbb {D}}|\Psi _{z} (a,s,p)| \; <\; 1$$.

### Theorem 2.10

[4, Theorem 5.3] Let

$$\begin{aligned} (s, p) = (\beta +{\overline{\beta }}p, p) = (\lambda _1+\lambda _2, \lambda _1\lambda _2)\in \Gamma , \end{aligned}$$

where $|\beta | \le 1$ and if $|p| = 1$ then $\beta = \frac{1}{2}s$. Let $a \in \mathbb {C}$. The following statements are equivalent:

1. $$(a, s, p) \in \overline{\mathcal {P}}$$;
2. $$|a| \le |1-\frac{\frac{1}{2}s{\overline{\beta }}}{1+\sqrt{1-|\beta |^2}}|$$;
3. $$|a| \le \frac{1}{2}|1-{\overline{\lambda }}_2\lambda _1| + \frac{1}{2}(1-|\lambda _1|^2)^{\frac{1}{2}}(1-|\lambda _2|^2)^{\frac{1}{2}}$$;
4. $|\Psi _z(a, s, p)| \le 1$ for all $z \in \mathbb {D}$, where $\Psi _z$ is defined by Eq. (2.2);
5. there exists $A \in \mathbb {C}^{2\times 2}$ such that $\Vert A\Vert \le 1$ and $\pi (A) = (a, s, p).$

## The Distinguished Boundary of $\mathcal {P}$

Let $\Omega$ be a domain in $\mathbb {C}^n$ with closure ${\overline{\Omega }}$ and let $A(\Omega )$ be the algebra of continuous scalar functions on ${\overline{\Omega }}$ that are holomorphic on $\Omega$. A *boundary* for $\Omega$ is a subset *C* of ${\overline{\Omega }}$ such that every function in $A(\Omega )$ attains its maximum modulus on *C*. Since $\overline{\mathcal {P}}$ is polynomially convex, there is a smallest closed boundary of $\mathcal {P}$, contained in all the closed boundaries of $\mathcal {P}$, called the distinguished boundary of $\mathcal {P}$ and denoted by $b\overline{\mathcal {P}}$. If there is a function $g \in A(\mathcal {P})$ and a point $u \in \overline{\mathcal {P}}$ such that $g(u)=1$ and |*g*(*x*)| 1 for all $x \in \overline{\mathcal {P}} \backslash \{u\}$, then *u* must belong to $b\overline{\mathcal {P}}$. Such a point *u* is called a *peak point* of $\overline{\mathcal {P}}$ and the function *g* a *peaking function* for *u*. Define

$$\begin{aligned} K_0 {\mathop {=}\limits ^{def }}\bigg \{(a, s, p) \in \mathbb {C}^3 : (s, p) \in b\Gamma , \ |a| = \sqrt{1-\frac{1}{4}|s|^2}\bigg \}. \end{aligned}$$

and

3.1 $$\begin{aligned} K_1 {\mathop {=}\limits ^{def }}\bigg \{(a, s, p) \in \mathbb {C}^3 : (s, p) \in b\Gamma , \ |a| \le \sqrt{1-\frac{1}{4}|s|^2}\bigg \}. \end{aligned}$$

### Proposition 3.1

[4, Proposition 8.3] The subsets $K_0$ and $K_1$ of $\overline{\mathcal {P}}$ are closed boundaries for $A(\mathcal {P})$.

### Theorem 3.2

[4, Theorem 8.4] For $x \in \mathbb {C}^3$, the following are equivalent:

1. $$x \in K_0$$;
2. *x* is a peak point of $\overline{\mathcal {P}}$;
3. $x \in b\overline{\mathcal {P}}$, the distinguished boundary of $\mathcal {P}$. Therefore,
  $$\begin{aligned} b\overline{\mathcal {P}} = \bigg \{(a, s, p) \in \mathbb {C}^3 : (s, p) \in b\Gamma , \ |a| = \sqrt{1-\frac{1}{4}|s|^2}\bigg \} \end{aligned}$$
  and so
  3.2 $$\begin{aligned} b\overline{\mathcal {P}} = \bigg \{(a, s, p) \in \mathbb {C}^3 : |s| \le 2, \ |p|=1, \ s = \overline{s}p \ and \ |a| = \sqrt{1-\frac{1}{4}|s|^2}\bigg \}.\nonumber \\ \end{aligned}$$

### Theorem 3.3

[4, Theorem 8.5] The distinguished boundary $b\overline{\mathcal {P}}$ is homeomorphic to

$$\begin{aligned} \{(\sqrt{1-x^2}w, x, \theta ) : -1 \le x \le 1, \ 0 \le \theta \le 2\pi , \ w \in \mathbb {T}\} \end{aligned}$$

with the two points $(\sqrt{1-x^2}w, x, 0)$ and $(\sqrt{1-x^2}w, -x, 2\pi )$ identified for every $w \in \mathbb {T}$ and $x \in [-1, 1]$.

## The Royal Variety of $\mathcal {P}$ and Aut $\mathcal {P}$

Recall that $\mathcal {P}=\pi (\mathbb {B}^{2\times 2})$ where $\pi :\mathbb {C}^{2\times 2}\rightarrow \mathbb {C}^3$ is defined as

4.1 $$\begin{aligned} \pi :A \mapsto (a_{21}, tr A, det A). \end{aligned}$$

We define the singular set of $\mathcal {P}=\pi (\mathbb {B}^{2\times 2})$ to be the image under $\pi$ of the set of critical points of $\pi$.

### Proposition 4.1

The singular set of the pentablock is $\mathcal {R}_{\mathcal {P}}=\{(0, s, p )\in \mathcal {P}: s^2=4p\}$.

### Proof

The set of critical points of $\pi$ is the set $\pi (\{A \in \mathbb {B}^{2\times 2}: \textbf{J}_{\pi }(A)$ is not of full rank}), where $\textbf{J}_{\pi }(A)$ is the Jacobian matrix of $\pi$.

The Jacobian matrix of $\pi$, $\textbf{J}_{\pi }(A)$, is defined by

$$\begin{aligned} \textbf{J}_{\pi }(A)= \begin{bmatrix} \dfrac{\partial \pi _1}{\partial a_{11}} &{} \dfrac{\partial \pi _1}{\partial a_{12}} &{} \dfrac{\partial \pi _1}{\partial a_{21}} &{} \dfrac{\partial \pi _1}{\partial a_{22}} \\ \\ \dfrac{\partial \pi _2}{\partial a_{11}} &{} \dfrac{\partial \pi _2}{\partial a_{12}} &{} \dfrac{\partial \pi _2}{\partial a_{21}} &{} \dfrac{\partial \pi _2}{\partial a_{22}} \\ \\ \dfrac{\partial \pi _3}{\partial a_{11}} &{} \dfrac{\partial \pi _3}{\partial a_{12}} &{} \dfrac{\partial \pi _3}{\partial a_{21}} &{} \dfrac{\partial \pi _3}{\partial a_{22}} &{} \end{bmatrix}\;\; \text {for} \;\; A= \begin{bmatrix} a_{11} &{} a_{12} \\ a_{21} &{} a_{22} \end{bmatrix} \in \mathbb {C}^{2 \times 2}. \end{aligned}$$

Thus,

$$\begin{aligned} \textbf{J}_\pi (A)= \begin{bmatrix} 0 &{} 0 &{} 1 &{} 0 \\ 1 &{} 0 &{} 0 &{} 1 \\ a_{22} &{} -a_{21} &{} -a_{12} &{} a_{11} \end{bmatrix}. \end{aligned}$$

Note that $\textbf{J}_{\pi }(A)$ is not of full rank if and only if rank $\textbf{J}_{\pi }(A) \le 2$. That means all $3\times 3$ minors of $\textbf{J}_{\pi }(A)$ are zero. Let us find all $3\times 3$ minors of $\textbf{J}_\pi (A)$.

$$\begin{aligned} \begin{vmatrix} 0&0&1\\ 1&0&0 \\ a_{22}&-a_{21}&-a_{12} \end{vmatrix}=-a_{21},&~~&\begin{vmatrix} 0&1&0\\0&0&1 \\ -a_{21}&-a_{12}&a_{11} \end{vmatrix}=-a_{21}, \\ \begin{vmatrix} 0&0&0\\1&0&1 \\ a_{22}&-a_{21}&a_{11} \end{vmatrix}=0,&~~&\begin{vmatrix} 0&1&0\\1&0&1 \\ a_{22}&-a_{12}&a_{11} \end{vmatrix}=-a_{11}+a_{22}. \end{aligned}$$

Thus $\textbf{J}_\pi (A)$ is not of full rank if and only if $a_{21}=0$ and $a_{11}=a_{22}$. Therefore,

$$\begin{aligned} \mathcal {R}_{\mathcal {P}} = \pi (\{A \in \mathbb {B}^{2\times 2}:\textbf{J}_\pi (A) \text { is not of full rank} \})= & {} \pi \Bigg ( A = \begin{bmatrix} a &{} * \\ 0 &{} a \end{bmatrix} \in \mathbb {B}^{2\times 2} \Bigg )\\= & {} \bigg \{ \Big (0, 2a, a^2 \Big ): a \in \mathbb {D}\bigg \}\\= & {} \bigg \{ \Big (0, s, p \Big ): (s, p)\in \mathbb {G}, s^2=4p\bigg \}. \end{aligned}$$

here $s=$ tr *A* and $p=$ det *A*. $\square$

### Remark 4.2

The singular set of the pentablock can be presented as

$$\begin{aligned} \mathcal {R}_{\mathcal {P}} = \{(0, s, p) \in \mathcal {P} : (s, p) \in \mathcal {R}_\Gamma \cap \mathcal {G}\}. \end{aligned}$$

By analogy with the established terminology for the symmetrized bidisc, we shall call the set

$$\begin{aligned} \mathcal {R}_{\overline{\mathcal {P}}}= \{(0, s, p) \in \mathbb {C}^3 : s^2= 4p\}. \end{aligned}$$

the *royal variety* of the pentablock.

### Lemma 4.3

Let $(a, s, p) \in b\overline{\mathcal {P}}$. Then the following conditions are equivalent:

(i) $$a = 0$$;
(ii) $$(a, s, p) \in b\overline{\mathcal {P}} \cap \mathcal {R}_{\overline{\mathcal {P}}}$$;
(iii) $$|s|=2$$.

### Proof

It easily follows from the definition of $\mathcal {R}_{\overline{\mathcal {P}}}$ and the formula (3.2) for $b\overline{\mathcal {P}}$. $\square$

**The automorphism group of** $\mathcal {P}$. Recall the known information on the automorphism group Aut $\mathcal {P}$ of $\mathcal {P}$ from [4]. For $w \in \mathbb {T}$ and $v \in Aut \ \mathbb {D}$, let

4.2 $$\begin{aligned} f_{wv}(a, s, p) = \Big (\frac{w\eta (1-|\alpha |^2)a}{1-{\overline{\alpha }}s+{\overline{\alpha }}^2p}, \tau _v(s, p) \Big ), \end{aligned}$$

where $v=\eta B_\alpha$ for $\alpha \in \mathbb {D}$, $\eta \in \mathbb {T}$, $B_\alpha (z) = \dfrac{z-\alpha }{1-{\overline{\alpha }}z}$ is a Blaschke factor and $\tau _v \in Aut \ \mathbb {G}$ is defined by

$$\begin{aligned} \tau _v(z+w, zw) = \big (v(z)+v(w), v(z)v(w)\big ). \end{aligned}$$

$$f_{wv}(a, s, p)$$fwv(a,s,p)

### Theorem 4.4

[4, Theorem 7.1] The maps $f_{wv}$, for $w \in \mathbb {T}$ and $v \in Aut \ \mathbb {D}$, constitute a group of automorphisms of $\mathcal {P}$ under composition. Each automorphism $f_{wv}$ extends analytically to a neighbourhood of $\overline{\mathcal {P}}$.

Moreover, for all $w_1, w_2 \in \mathbb {T}$, $v_1, v_2 \in Aut \ \mathbb {D}$,

$$\begin{aligned} f_{w_1v_1} \circ f_{w_2v_2} = f_{(w_1w_2)(v_1\circ v_2)}, \end{aligned}$$

and, for all $w \in \mathbb {T}$, $v \in Aut \ \mathbb {D}$,

$$\begin{aligned} (f_{wv})^{-1} = f_{\overline{w}v^{-1}}. \end{aligned}$$

Kosiński proved in [26] that the set $\{f_{wv}:w \in \mathbb {T}, v \in Aut \ \mathbb {D}\}$ is the full group of automorphisms of $\mathcal {P}$.

### Lemma 4.5

$\mathcal {R}_{\overline{\mathcal {P}}} \cap \mathcal {P}$ is invariant under Aut $\mathcal {P}$.

### Proof

Every element of $\mathcal {R}_{\overline{\mathcal {P}}} \cap \mathcal {P}$ is of the form $(0, s, p) \in \mathcal {P}$, where $s^2 = 4p$. It is easy to see that, for every element $f_{wv}$ of Aut $\mathcal {P}$ given by Eq. (4.2),

$$\begin{aligned} f_{wv}(0, s, p) = \Big (0, \tau _v(s, p) \Big ). \end{aligned}$$

Since $\tau _v \in Aut \ \mathbb {G}$ and $(s, p)\in \mathcal {R}_\Gamma \cap \mathbb {G}$, by Lemma 2.4, $\tau _v(s, p) \in \mathcal {R}_\Gamma \cap \mathbb {G}$. Therefore, $f_{wv}(0, s, p) \in \mathcal {R}_{\overline{\mathcal {P}}}.$ $\square$

For any domain *U* in $\mathbb {C}^{n}$, $\text {Hol}(\mathbb {D}, U)$ denotes the space of analytic functions from $\mathbb {D}$ to *U*.

### Definition 4.6

Let *U* be a domain in $\mathbb {C}^n$ and let $\mathcal {D} \subset U$. We say $\mathcal {D}$ is a complex geodesic in *U* if there exists a function $k \in Hol (\mathbb {D}, U)$ and a function $C \in Hol (U, \mathbb {D})$ such that $C \circ k = \mathrm id_{\mathbb {D}}$ and $\mathcal {D} = k(\mathbb {D})$.

For a geometric classification of complex geodesics in the symmetrized bidisc $\mathbb {G}$, see [7]. We define, for $\omega \in \mathbb {T}$, the rational function $\Phi _\omega$ of two variables by

$$\begin{aligned} \Phi _\omega (s, p) = \frac{2\omega p-s}{2-\omega s}, \; \; \text {where} \;\; \omega s \ne 2 . \end{aligned}$$

In the function theory and geometry of $\mathbb {G}$ much depends on the properties of these functions $\Phi _\omega ,\; \omega \in \mathbb {T}$, see [14].

### Lemma 4.7

$\mathcal {R}_{\overline{\mathcal {P}}} \cap \mathcal {P}$ is a complex geodesic in $\mathcal {P}$.

### Proof

Define the analytic functions *k* and *c* by

$$\begin{aligned} k:\mathbb {D}\rightarrow \mathcal {P}, \; k(\lambda ) = (0, -2\lambda , \lambda ^2) \end{aligned}$$

and

$$\begin{aligned} c:\mathcal {P} \rightarrow \mathbb {D}, \; c(a, s, p) = \Phi _\omega (s, p) = \frac{2\omega p-s}{2-\omega s}, \; \; \text {where} \;\; \omega \in \mathbb {T}. \end{aligned}$$

For $$\lambda \in \mathbb {D}$$,

$$\begin{aligned} (c \; \circ \; k)(\lambda ) = c(k(\lambda )) = c(0, -2\lambda , \lambda ^2) = \frac{2\omega \lambda ^2+2\lambda }{2+2\omega \lambda } = \lambda , \end{aligned}$$

which means $c \; \circ \; k = \mathrm id_{\mathbb {D}}.$ By definition of $\mathcal {R}_{\overline{\mathcal {P}}}$, it is easy to see that $\mathcal {R}_{\overline{\mathcal {P}}} \cap \mathcal {P} = k(\mathbb {D})$. Therefore, $\mathcal {R}_{\overline{\mathcal {P}}} \cap \mathcal {P}$ is a complex geodesic in $\mathcal {P}$. $\square$

## Examples of $\overline{\mathcal {P}}$-Inner Functions

Descriptions of inner and outer functions in $H^\infty (\mathbb {D})$ and properties of inner and outer functions can be found [29, Chapter III]. Here $H^\infty (\mathbb {D})$ is the space of holomorphic functions *u* on $\mathbb {D}$ such that the corresponding norm

$$\begin{aligned} \Vert u\Vert _\infty = \sup \limits _{\lambda \in \mathbb {D}} |u(\lambda )| \end{aligned}$$

is finite.

### Definition 5.1

An inner function is an analytic map $f:\mathbb {D} \rightarrow \overline{\mathbb {D}}$ such that the radial limit

$$\begin{aligned} \lim _{r \rightarrow 1^{-}}f(r\lambda ) \end{aligned}$$

exists and belongs to $\mathbb {T}$ for almost all $\lambda \in \mathbb {T}$ with respect to Lebesgue measure.

It is well known that the rational inner functions on $\mathbb {D}$ are precisely the finite Blaschke products. One can see that the only functions which are at the same time inner and outer are the constant functions of modulus 1.

### Definition 5.2

A $\overline{\mathcal {P}}$-inner or penta-inner function is an analytic map $f:\mathbb {D}\rightarrow \overline{\mathcal {P}}$ such that the radial limit

$$\begin{aligned} \lim _{r \rightarrow 1^{-}}f(r\lambda ) \end{aligned}$$

exists and belongs to $b\overline{\mathcal {P}}$ for almost all $\lambda \in \mathbb {T}$ with respect to Lebesgue measure.

By Fatou’s Theorem, the $\lim _{r \rightarrow 1^{-}}f(r\lambda )$ exists for almost all $\lambda \in \mathbb {T}$.

### Remark 5.3

Let $f:\mathbb {D}\rightarrow \overline{\mathcal {P}}$ be a rational $\overline{\mathcal {P}}$-inner function. Since *f* is rational and bounded on $\mathbb {D}$ it has no poles in $\overline{\mathbb {D}}$ and hence *f* is continuous on $\overline{\mathbb {D}}$. Thus one can consider the continuous function

$$\begin{aligned} {\tilde{f}}:\mathbb {T}\rightarrow b\overline{\mathcal {P}}, \text {where} {\tilde{f}}(\lambda )=\lim _{r \rightarrow 1^-}f(r\lambda ) \text { for all } \lambda \in \mathbb {T}. \end{aligned}$$

### Example 5.4

Let us consider an example of an analytic function $f:\mathbb {D}\rightarrow \overline{\mathcal {P}}$. Consider the analytic map $h:\mathbb {D}\rightarrow \mathbb {B}^{2\times 2}$ defined by

5.1 $$\begin{aligned} h(\lambda )= \begin{bmatrix} \varphi (\lambda ) &{} 0 \\ 0 &{} \psi (\lambda ) \end{bmatrix} \ \ \ \text {for} \ \lambda \in \mathbb {D}, \end{aligned}$$

where $\varphi , \psi \in H^\infty (\mathbb {D})$ are non-constant inner functions. Note that

$$\begin{aligned} \Vert h(\lambda )\Vert = \max \{|\varphi (\lambda )|,|\psi (\lambda )|\} \; <\; 1\; \text { for} \; \lambda \in \mathbb {D}. \end{aligned}$$

For all $\lambda \in \mathbb {D}$, let

$$\begin{aligned} f(\lambda ) = \pi (h(\lambda ))=(0, {\text {tr}}h(\lambda ), \det h(\lambda )). \end{aligned}$$

*Let us show that the function* *f* *is a* $\overline{\mathcal {P}}$ *-inner function only when* $\varphi = \psi$.

Let, for $\lambda \in \mathbb {D}, \; a(\lambda ) = 0, \; s(\lambda ) = {\text {tr}}h(\lambda ) = \varphi (\lambda )+\psi (\lambda )$ and $p(\lambda ) = \det h(\lambda ) = \varphi (\lambda )\psi (\lambda )$. Clearly $f=(a, s, p):\mathbb {D}\rightarrow \overline{\mathcal {P}}$ is an analytic function.

To prove that *f* is $\overline{\mathcal {P}}$-inner, we need to check that $f(\lambda ) \in b\overline{\mathcal {P}}$ for almost every $\lambda \in \mathbb {T}$, that is, $\big (s(\lambda ), p(\lambda )\big ) \in b\Gamma$ and $\sqrt{1-\frac{1}{4}|s(\lambda )|^2} = 0$ for almost every $\lambda \in \mathbb {T}$. Note that, for almost every $\lambda \in \mathbb {T}$,

$$\begin{aligned} |p(\lambda )| = |\varphi (\lambda )\psi (\lambda )| = |\varphi (\lambda )||\psi (\lambda )| = 1, \ \text { since } \ |\varphi (\lambda )| = 1 \text { and } \ |\psi (\lambda )| = 1, \\ |s(\lambda )| = |\varphi (\lambda )+\psi (\lambda )| \le |\varphi (\lambda )|+|\psi (\lambda )| = 2, \end{aligned}$$

and

$$\begin{aligned} (\overline{s}p)(\lambda )= & {} \big (\overline{\varphi (\lambda )+\psi (\lambda )}\big )\big (\varphi (\lambda ) \psi (\lambda )\big ) = \varphi (\lambda )\overline{\varphi (\lambda )}\psi (\lambda ) + \varphi (\lambda )\psi (\lambda )\overline{\psi (\lambda )} \\= & {} |\varphi (\lambda )|^2 \psi (\lambda ) + \varphi (\lambda )|\psi (\lambda )|^2 = \varphi (\lambda )+\psi (\lambda ) = s(\lambda ). \end{aligned}$$

Hence for almost every $\lambda \in \mathbb {T}, \ |p(\lambda )| = 1, \ |s(\lambda )|\le 2$ and $(\overline{s}p)(\lambda ) = s(\lambda )$, and so $\big ({\text {tr}}h(\lambda ), \det h(\lambda )\big ) \in b\Gamma$. Now, for almost every $\lambda \in \mathbb {T}$,

$$\begin{aligned} 1-\frac{1}{4}|s(\lambda )|^2= & {} 1-\frac{1}{4}|\varphi (\lambda )+\psi (\lambda )|^2 = 1-\frac{1}{4}\Big (\big (\varphi (\lambda )+\psi (\lambda )\big )\big (\overline{\varphi (\lambda )+\psi (\lambda )}\big )\Big ) \\= & {} 1-\frac{1}{4}\Big (1+1+2 \textrm{Re}\big (\varphi (\lambda )\overline{\psi (\lambda )}\big )\Big ) = \frac{1}{2}-\frac{1}{2}\textrm{Im}\big (i\varphi (\lambda )\overline{\psi (\lambda )}\big ). \end{aligned}$$

Hence $|a|=\sqrt{1-\frac{1}{4}|s|^2}$ almost everywhere on $\mathbb {T}$ if and only if

$$\begin{aligned} \frac{1}{2}-\frac{1}{2} \textrm{Im} \big (i\varphi (\lambda )\overline{\psi (\lambda )}\big ) = 0, \text { for almost every }\lambda \in \mathbb {T}, \end{aligned}$$

if and only if $\textrm{Im}(i\varphi (\lambda )\overline{\psi (\lambda )}) = 1 \text { for almost every }\lambda \in \mathbb {T}.$ Therefore, $|a|=\sqrt{1-\frac{1}{4}|s|^2}$ almost everywhere on $\mathbb {T}$ if and only if $\varphi (\lambda )\overline{\psi (\lambda )} = 1$ almost everywhere on $\mathbb {T}$, and so, $\varphi (\lambda ) = \psi (\lambda )$ almost everywhere on $\mathbb {T}$. Thus the function *f* is a $\overline{\mathcal {P}}$-inner function only when $\varphi = \psi$, and so $f = (0, 2 \varphi , \varphi ^2)$.

### Example 5.5

Let $h_1 : \mathbb {D} \rightarrow \mathbb {C}^{2 \times 2}$ be defined by $h_1=Uh$, where *h* is defined by Eq. (5.1) and

$$\begin{aligned} U= \begin{bmatrix} \dfrac{1}{\sqrt{2}} &{} \dfrac{1}{\sqrt{2}} \\ \dfrac{1}{\sqrt{2}}i &{} -\dfrac{1}{\sqrt{2}}i \end{bmatrix}. \end{aligned}$$

Note that *U* is a unitary matrix. Then, for $\lambda \in \mathbb {D}$,

$$\begin{aligned} h_1(\lambda )= & {} Uh(\lambda ) \\= & {} \frac{1}{\sqrt{2}} \begin{bmatrix} \varphi (\lambda ) &{} \psi (\lambda ) \\ i\varphi (\lambda ) &{} -i\psi (\lambda ) \end{bmatrix}, \end{aligned}$$

and, for all $\lambda \in \mathbb {D}$,

$$\begin{aligned} \Vert h_1(\lambda )\Vert \le \Vert U\Vert \Vert h(\lambda )\Vert =\Vert h(\lambda )\Vert =\max \{|\varphi (\lambda )|, |\psi (\lambda )|\} < 1. \end{aligned}$$

Hence $h_1(\lambda ) \in \mathbb {B}^{2\times 2}$ for all $\lambda \in \mathbb {D}$. Define $f_1=\pi \circ h_1$ on $\mathbb {D}$. Then, for $\lambda \in \mathbb {D}$,

5.2 $$\begin{aligned} f_1(\lambda )&= \pi (h_1(\lambda )) \nonumber \\&= \pi \bigg (\frac{1}{\sqrt{2}} \begin{bmatrix} \varphi (\lambda ) &{} \psi (\lambda ) \\ i\varphi (\lambda ) &{} -i\psi (\lambda ) \end{bmatrix}\bigg ) \nonumber \\&= \bigg (\frac{i\varphi (\lambda )}{\sqrt{2}}, \frac{\varphi (\lambda )-i\psi (\lambda )}{\sqrt{2}}, -i\varphi (\lambda )\psi (\lambda )\bigg ). \end{aligned}$$

Clearly, $f_1:\mathbb {D}\rightarrow \overline{\mathcal {P}}$ is an analytic function since $\varphi , \psi$ are analytic on $\mathbb {D}$.

*Let us show that* $f_1$ *is a* $\overline{\mathcal {P}}$ *-inner function if and only if *$\varphi = i\psi$.

Let us check when the function $f_1$ is $\overline{\mathcal {P}}$-inner. We need to find conditions when $f_1$ maps $\mathbb {T}$ into the distinguished boundary $b\overline{\mathcal {P}}$ of $\mathcal {P}$. Since $\varphi$, $\psi$ are inner functions, they have unit modulus almost everywhere on $\mathbb {T}$. Thus, for $s = \dfrac{\varphi -i\psi }{\sqrt{2}}$, $p = -i\varphi \psi$ and for almost every $\lambda \in \mathbb {T}$,

$$\begin{aligned} |p(\lambda )|= & {} |-i\varphi (\lambda )\psi (\lambda )|=|\varphi (\lambda )||\psi (\lambda )|=1.\\ |s(\lambda )|= & {} \bigg |\frac{\varphi (\lambda )-i\psi (\lambda )}{\sqrt{2}}\bigg | \le \frac{|\varphi (\lambda )|+|-i\psi (\lambda )|}{\sqrt{2}}=\frac{2}{\sqrt{2}}.\\ (\overline{s}p)(\lambda )= & {} \frac{\overline{\varphi (\lambda )-i\psi (\lambda )}}{\sqrt{2}}(-i\varphi (\lambda )\psi (\lambda )) \\= & {} \frac{-i|\varphi (\lambda )|^2 \psi (\lambda )+\varphi (\lambda )|\psi (\lambda )|^2}{\sqrt{2}} = \frac{\varphi (\lambda )-i\psi (\lambda )}{\sqrt{2}}=s(\lambda ). \end{aligned}$$

Therefore, for almost every $\lambda \in \mathbb {T}, |p(\lambda )| = 1$, $|s(\lambda )|\le 2$ and $(\overline{s}p)(\lambda ) = s(\lambda )$ and so $(s(\lambda ), p(\lambda )) \in b\Gamma$. Finally,

$$\begin{aligned} \sqrt{1-\frac{1}{4}|s(\lambda )|^2}= & {} \sqrt{1-\frac{1}{4} \bigg (\frac{1}{2}|\varphi (\lambda )-i \psi (\lambda )|^2\bigg )} \\= & {} \sqrt{1-\frac{1}{8}\bigg (1+1+2 \textrm{Re} (\varphi (\lambda ) i \overline{\psi (\lambda )})\bigg )} = \frac{1}{2} \sqrt{3+\textrm{Im} (\varphi (\lambda )\overline{\psi (\lambda )})}. \end{aligned}$$

We want $|a|=\sqrt{1-\frac{1}{4}|s|^2}$ almost everywhere on $\mathbb {T}$, that is, for almost every $\lambda \in \mathbb {T}$,

$$\begin{aligned} \dfrac{1}{\sqrt{2}} = \dfrac{|\varphi (\lambda )|}{\sqrt{2}} = \dfrac{1}{2} \sqrt{3+\textrm{Im} (\varphi (\lambda )\overline{\psi (\lambda )})}. \end{aligned}$$

Hence $|a|=\sqrt{1-\frac{1}{4}|s|^2}$ almost everywhere on $\mathbb {T}$ if and only if $\sqrt{3+\textrm{Im} (\varphi (\lambda )\overline{\psi (\lambda )})} = \sqrt{2}$ for almost every $\lambda \in \mathbb {T}$, or equivalently, $\varphi (\lambda ) = -i\psi (\lambda )$ for almost every $\lambda \in \mathbb {T}$. Thus $f_1$ given by Eq. (5.2) is a $\overline{\mathcal {P}}$-inner function if and only if $\varphi = -i\psi$. In this case $f_1= \bigg (\frac{i\varphi }{\sqrt{2}}, \sqrt{2} \varphi , \varphi ^2\bigg )$.

### Example 5.6

Let *v*, $\varphi$ and $\psi$ be inner functions on $\mathbb {D}$. Consider the functions

$$\begin{aligned} V(\lambda ) = \frac{1}{\sqrt{2}}\begin{bmatrix} 1 &{} v \\ -1 &{} v \end{bmatrix}(\lambda ) \ \text { and } \ h(\lambda ) = \begin{bmatrix} \varphi &{} 0 \\ 0 &{} \psi \end{bmatrix}(\lambda ),\; \text { for }\; \lambda \in \mathbb {D}. \end{aligned}$$

Define

$$\begin{aligned} U(\lambda )= & {} (V^* h V)(\lambda )= \frac{1}{\sqrt{2}} \begin{bmatrix} 1 &{} -1 \\ \overline{v} &{} \overline{v} \end{bmatrix} \begin{bmatrix} \varphi &{} 0 \\ 0 &{} \psi \end{bmatrix} \frac{1}{\sqrt{2}} \begin{bmatrix} 1 &{} v \\ -1 &{} v \end{bmatrix}(\lambda ) \\= & {} \frac{1}{2} \begin{bmatrix} \varphi + \psi &{} (\varphi - \psi )v \\ (\varphi - \psi )\overline{v} &{} \varphi + \psi \end{bmatrix}(\lambda ), \ \text { for } \lambda \in \mathbb {D}. \end{aligned}$$

Note that, for all $\lambda \in \mathbb {D}$,

$$\begin{aligned} \Vert V^*(\lambda )\Vert = \left\| \begin{bmatrix} 1 &{} 0 \\ 0 &{} \overline{v}(\lambda ) \end{bmatrix} \frac{1}{\sqrt{2}} \begin{bmatrix} 1 &{} -1 \\ 1 &{} 1 \end{bmatrix} \right\| \le 1, \end{aligned}$$

since $$ \frac{1}{\sqrt{2}}\begin{bmatrix} 1 &{} -1 \\ 1 &{} 1 \end{bmatrix}$$ is unitary and $| \overline{v}(\lambda ) | \le 1$ on $\mathbb {D}$. Hence

$$\begin{aligned} \Vert U(\lambda )\Vert \le \Vert V(\lambda )\Vert ^2 \Vert h(\lambda )\Vert < 1 \ \text {for } \lambda \in \mathbb {D}. \end{aligned}$$

Define $f:\mathbb {D} \rightarrow \overline{\mathcal {P}}$ by $f=\pi \circ U$. Then, for $\lambda \in \mathbb {D}$,

5.3 $$\begin{aligned} f(\lambda ) = \pi \circ U(\lambda ) = \Big (\frac{1}{2}(\varphi - \psi )\overline{v}, \varphi + \psi , \frac{1}{4}\big ((\varphi + \psi )^2-(\varphi - \psi )^2|v|^2\big ) \Big )(\lambda ).\nonumber \\ \end{aligned}$$

Note that *f* is analytic on $\mathbb {D}$ if and only if *v* is constant or $\varphi = \psi$.

*Let us show that f* *is a* $\overline{\mathcal {P}}$*-inner function if and only if* *v* *is constant or* $\varphi = \psi$.

**Case 1** Suppose *v* is constant. As *v* is inner, $|v|=1$. Let us check that $f:\mathbb {D} \rightarrow \overline{\mathcal {P}}$ is $\overline{\mathcal {P}}$-inner, that is, $f(\mathbb {T}) \subset b\overline{\mathcal {P}}$. Note, for almost all $\lambda \in \mathbb {T}$, $\det U(\lambda ) = (\varphi \psi )(\lambda )$ and $(\varphi +\psi , \varphi \psi )(\lambda ) \in b\Gamma$ as in Example 5.4.

$$\begin{aligned} |a|^2 = \dfrac{1}{4}|\varphi - \psi |^2 = \dfrac{1}{4} \Big (1+1-2 \textrm{Re} ({\overline{\varphi }}\psi ) \Big ) = \dfrac{1}{2}-\dfrac{1}{2} \textrm{Re} ({\overline{\varphi }}\psi ) \text { almost everywhere on } \mathbb {T}. \\ 1 - \frac{1}{4} |s|^2 = 1 - \frac{1}{4}|\varphi + \psi |^2 = 1 - \frac{1}{4} \Big (1 + 1 + 2 \textrm{Re}({\overline{\varphi }}\psi ) \Big ) = 1-\frac{1}{2} \textrm{Re} ({\overline{\varphi }}\psi ) = |a|^2. \end{aligned}$$

Thus $|a|^2 = 1-\frac{1}{4}|s|^2$ almost everywhere on $\mathbb {T}$, and so, *f* given by Eq. (5.3) is a $\overline{\mathcal {P}}$-inner function if *v* is constant. Since $|v|=1$, $f = \Big (\frac{1}{2}(\varphi - \psi )\overline{v}, \varphi + \psi , \varphi \psi \Big )$.

**Case 2** Suppose $\varphi = \psi$. Then

$$\begin{aligned} f = \Big (0, 2\varphi , \frac{1}{4}(2\varphi )^2\Big ) = (0, 2\varphi , \varphi ^2). \end{aligned}$$

We have shown in Example 5.4 that $f= (0, 2\varphi , \varphi ^2)$ is a $\overline{\mathcal {P}}$-inner function.

### Example 5.7

Define the function $x(\lambda ) = (\lambda ^m, 0, \lambda ): \mathbb {D}\rightarrow \overline{\mathcal {P}}$. First we need to show that for all $\lambda \in \mathbb {D}, \; x(\lambda ) \in \overline{\mathcal {P}}$. *Let us show that* *x* *is a rational* $\overline{\mathcal {P}}$-*inner function.*

By Proposition 2.2, $(s, p) \in \Gamma$ if and only if

$$\begin{aligned} |s|\le 2 \ \text { and } |s-\overline{s}p|\le 1-|p|^2. \end{aligned}$$

It is easy to see that $(0, \lambda ) \in \Gamma$. By Theorem 2.10, if $(s, p) \in \Gamma$, then for $a \in \mathbb {C}, (a, s, p) \in \overline{\mathcal {P}}$ if and only if

5.4 $$\begin{aligned} |a| \le \left| 1-\frac{\frac{1}{2}s{\overline{\beta }}}{1+\sqrt{1-|\beta |^2}}\right| , \ \text { where } \ \beta =\frac{s-\overline{s}p}{1-|p|^2}. \end{aligned}$$

In the case $s = 0$, Eq. (5.4) is equivalent to $|a| \le 1$. Note that $x(\lambda ) = \big (a(\lambda ), s(\lambda ), p(\lambda )\big ) = (\lambda ^m, 0, \lambda ), \; \lambda \in \mathbb {D}, \text { is analytic in } \mathbb {D}$, and

$$\begin{aligned} |a(\lambda )| = |\lambda ^m| \le 1, \ \text { for all } \ \lambda \in \mathbb {D}. \end{aligned}$$

Thus for $$\lambda \in \mathbb {D}, \ x(\lambda ) \in \overline{\mathcal {P}}$$.

Now, let us check that *x* maps $\mathbb {T}$ into the distinguished boundary $b\overline{\mathcal {P}}$ of $\mathcal {P}$. For all $\lambda \in \mathbb {T}$,

$$\begin{aligned} |p(\lambda )| = |\lambda | = 1, \ |s(\lambda )| = |0| \le 2, \\ (\overline{s}p)(\lambda ) = 0 = s(\lambda ) \ \text { and } \\ |a(\lambda )| = |\lambda ^m| = \sqrt{1-\frac{1}{4}|s(\lambda )|^2} = 1. \end{aligned}$$

Therefore, for every $\lambda \in \mathbb {T}$, $x(\lambda ) \in b\overline{\mathcal {P}}$ and hence *x* is a rational $\overline{\mathcal {P}}$-inner function.

### Example 5.8

For $\lambda \in \mathbb {D}$, define the function $x(\lambda ) = \big (a(\lambda ), s(\lambda ), p(\lambda )\big )= (\lambda , 0, \lambda ^n)$. As in the previous example, for all $\lambda \in \mathbb {D}$, by Proposition 2.2, $(0, \lambda ^n) \in \Gamma$. For $a \in \mathbb {C}$, we want

5.5 $$\begin{aligned} |a| \le |1-\frac{\frac{1}{2}s{\overline{\beta }}}{1+\sqrt{1-|\beta |^2}}|. \end{aligned}$$

Since $s = 0$, the condition (5.5) is equivalent to $|a| \le 1$. Note that

$$\begin{aligned} |a(\lambda )| = |\lambda | \le 1, \text { for all } \ \lambda \in \mathbb {D}. \end{aligned}$$

Thus, by Theorem 2.10, for $\lambda \in \mathbb {D}, \ x(\lambda ) \in \overline{\mathcal {P}}$. *Let us show that* *x* *is a rational* $\overline{\mathcal {P}}$- *inner function.*

Let us check that *x* maps $\mathbb {T}$ into the distinguished boundary $b\overline{\mathcal {P}}$ of $\mathcal {P}$. For all $\lambda \in \mathbb {T}$,

$$\begin{aligned} |p(\lambda )| = |\lambda ^n| = 1, \ |s(\lambda )| = |0| \le 2, \\ (\overline{s}p)(\lambda ) = 0 = s(\lambda ) \ \ \text {and } \\ |a(\lambda )| = |\lambda | = \sqrt{1-\frac{1}{4}|s(\lambda )|^2} = 1. \end{aligned}$$

Therefore, for every $\lambda \in \mathbb {T}$, $x(\lambda ) \in b\overline{\mathcal {P}}$ and hence *x* is a rational $\overline{\mathcal {P}}$-inner function.

## Some Properties of Analytic Functions $x:\mathbb {D}\rightarrow \overline{\mathcal {P}}$

In this section and in Sect. 7, we will show that there are close relations between $\overline{\mathcal {P}}$-inner functions and $\Gamma$-inner functions. Recall that the $\Gamma$-inner functions were first mentioned in [13]. A good understanding of rational $\Gamma$-inner functions will play an important part in any future solution of the finite interpolation problem for $\text {Hol}(\mathbb {D}, \Gamma )$, since such a problem has a solution if and only if it has a rational $\Gamma$-inner solution (see, for example, [18, Theorem 4] and [3, Theorem 8.1]). The rational $\Gamma$-inner functions were classified in [2]. Algebraic and geometric aspects of rational $\Gamma$-inner functions were presented in [6].

### Definition 6.1

A $\Gamma$-inner function is an *analytic function* $h:\mathbb {D} \rightarrow \Gamma$ such that the radial limit

6.1 $$\begin{aligned} \lim _{r \rightarrow 1^-} h(r \lambda ) \end{aligned}$$

exists and belongs to $b\Gamma$ for almost all $\lambda \in \mathbb {T}$ with respect to Lebesgue measure.

### Lemma 6.2

(i) Let $x = (a, s, p) : \mathbb {D} \rightarrow \overline{\mathcal {P}}$ be an analytic function. Then $h = (s, p) : \mathbb {D} \rightarrow \Gamma$ is an analytic function.
(ii) Let $x = (a, s, p) : \mathbb {D} \rightarrow \overline{\mathcal {P}}$ be a $\overline{\mathcal {P}}$-inner function. Then $h = (s, p) : \mathbb {D} \rightarrow \Gamma$ is a $\Gamma$-inner function.

### Proof

(i) By assumption, $x = (a, s, p)$ is analytic on $\mathbb {D}$ and for all $\lambda \in \mathbb {D}$, $x(\lambda ) = (a(\lambda ), s(\lambda ), p(\lambda )) \in \overline{\mathcal {P}}$. By Remark 2.5, for all $\lambda \in \mathbb {D}$, $(s(\lambda ), p(\lambda )) \in \Gamma$. Thus $h = (s, p):\mathbb {D} \rightarrow \Gamma$, where $h(\lambda ) = (s(\lambda ), p(\lambda ))$, for $\lambda \in \mathbb {D}$, is well defined and analytic from $\mathbb {D}$ to $\Gamma$.
(ii) By assumption $x = (a, s, p):\mathbb {D} \rightarrow \overline{\mathcal {P}}$ is a penta-inner function, and so, for almost all $\lambda \in \mathbb {T}$, $x(\lambda ) \in b\overline{\mathcal {P}}$. Recall $b\overline{\mathcal {P}} = \Big \{(a, s, p) \in \mathbb {C}^3 : (s, p) \in b\Gamma , \; |a| = \sqrt{1-\frac{1}{4}|s|^2}\Big \}$. By Theorem 3.2, for almost all $\lambda \in \mathbb {T}$, $h(\lambda ) = \big (s(\lambda ), p(\lambda )\big ) \in b\Gamma$. Hence *h* is a $\Gamma$-inner function.

$$\square $$

Recall that, by Proposition 3.1, $K_1 = \bigg \{(a, s, p) \in \overline{\mathcal {P}} : (s, p) \in b\Gamma , \ |a| \le \sqrt{1-\frac{1}{4}|s|^2}\bigg \}$ is a closed boundary of $A(\mathcal {P})$.

### Proposition 6.3

(i) Let $h = (s, p) : \mathbb {D} \rightarrow \Gamma$ be an analytic function. Then $x = (0, s, p)$ is an analytic function from $\mathbb {D}$ to $\overline{\mathcal {P}}$. (ii) Let $h = (s, p) : \mathbb {D} \rightarrow \Gamma$ be a $\Gamma$-inner function. Then $x = (0, s, p):\mathbb {D} \rightarrow \overline{\mathcal {P}}$ is an analytic function such that, for almost all $\lambda \in \mathbb {T}, \; x(\lambda ) \in K_1$.

### Proof

(i) It follows from Theorem 2.10 that, for all $\lambda \in \mathbb {D}, \big (0, s(\lambda ), p(\lambda )\big ) \in \overline{\mathcal {P}}$.
(ii) Suppose that *h* is a $\Gamma$-inner function. By Proposition 2.2, $|p(\lambda )| = 1, \ |s(\lambda )| \le 2 \ \text { and } \ (\overline{s}p)(\lambda ) = s(\lambda )$, for almost all $\lambda \in \mathbb {T}$. Since $a = 0 \text { and } \sqrt{1-\frac{1}{4}|s(\lambda )|^2} \ge 0$ for almost all $\lambda \in \mathbb {T}$, $x(\mathbb {T}) \subset K_1$.

$$\square $$

### Proposition 6.4

Let $x = (a, s, p)$ be a $\overline{\mathcal {P}}$-inner function. Let $a_{\textrm{in}} \; a_\textrm{out}$ be the inner-outer factorization of *a*. Then ${\widetilde{x}} = (a_\textrm{out}, s, p)$ is a $\overline{\mathcal {P}}$-inner function.

### Proof

By assumption $x = (a, s, p)$ is a $\overline{\mathcal {P}}$-inner function. Hence, for each $\lambda \in \mathbb {D}$, $\;(a(\lambda ), s(\lambda ), p(\lambda )) \in \overline{\mathcal {P}}$. By Theorem 2.10, for all $\lambda \in \mathbb {D}$, $|\Psi _z(a(\lambda ), s(\lambda ), p(\lambda ))| \le 1$ for all $z \in \mathbb {D}.$ Thus

$$\begin{aligned} \left| \frac{a(\lambda )(1-|z|^2)}{1-s(\lambda )z+p(\lambda )z^2}\right| \le 1, \; \text { for all } \lambda , z \in \mathbb {D}. \end{aligned}$$

Recall that $a=a_{\textrm{in}}a_{\textrm{out}}$, where $a_{\textrm{in}}$ is inner, and so $|a_{\textrm{in}}(\lambda )| = 1$ for almost all $\lambda \in \mathbb {T}$. Therefore, for every $z \in \mathbb {D}$, and, for almost all $\lambda \in \mathbb {T}$,

$$\begin{aligned} \left| a_{\textrm{in}}(\lambda )\frac{a_{\textrm{out}}(\lambda )(1-|z|^2)}{1-s(\lambda )z+p(\lambda )z^2}\right| = \left| \frac{a_{\textrm{out}}(\lambda )(1-|z|^2)}{1-s(\lambda )z+p(\lambda )z^2}\right| \le 1. \end{aligned}$$

Note that, for every $z \in \mathbb {D}$, the function

$$\begin{aligned} \lambda \mapsto \frac{a_{\textrm{out}}(\lambda )(1-|z|^2)}{1-s(\lambda )z+p(\lambda )z^2} \end{aligned}$$

is analytic on $\mathbb {D}.$ By the maximum principle for analytic functions, for every $z \in \mathbb {D}$,

$$\begin{aligned} \left| \frac{a_{\textrm{out}}(\lambda )(1-|z|^2)}{1-s(\lambda )z+p(\lambda )z^2}\right| \le 1, \; \text { for all } \lambda \in \mathbb {D}. \end{aligned}$$

Hence, by Theorem 2.10, for each $\lambda \in \mathbb {D}, \; (a_{\textrm{out}}(\lambda ), s(\lambda ), p(\lambda )) \in \overline{\mathcal {P}}$. Therefore, ${\widetilde{x}} = (a_{\textrm{out}}, s, p) \in$ Hol$(\mathbb {D}, \overline{\mathcal {P}})$.

To prove the statement of the proposition, we must show that, for almost all $\lambda \in \mathbb {T}$, ${\widetilde{x}}(\lambda ) = (a_{\textrm{out}}(\lambda ), s(\lambda ), p(\lambda )) \in b\overline{\mathcal {P}}$. Recall that

$$\begin{aligned} b\overline{\mathcal {P}} = \left\{ (a, s, p) \in \mathbb {C}^3 : (s, p) \in b\Gamma , \; |a|=\sqrt{1-\frac{1}{4}|s|^2}\right\} . \end{aligned}$$

By Lemma 6.2, for almost all $\lambda \in \mathbb {T}$, $(s(\lambda ), p(\lambda )) \in b\Gamma$. Since $x = (a, s, p)$ is a $\overline{\mathcal {P}}$-inner function, we have, for almost all $\lambda \in \mathbb {T}$,

$$\begin{aligned} |a(\lambda )| = \sqrt{1-\frac{1}{4}|s(\lambda )|^2}. \end{aligned}$$

Since $a_{\textrm{in}}a_{\textrm{out}} = a$ is the inner-outer factorization of *a* and $|a_{\textrm{in}}(\lambda )| = 1$ for almost all $\lambda \in \mathbb {T}$,

$$\begin{aligned} |a_{\textrm{out}}(\lambda )| = \sqrt{1-\frac{1}{4}|s(\lambda )|^2} \; \text { for almost all } \lambda \in \mathbb {T}. \end{aligned}$$

Therefore, ${\widetilde{x}} = (a_{\textrm{out}}, s, p)$ is a $\overline{\mathcal {P}}$-inner function. $\square$

## Connections Between Rational $\Gamma$-Inner and Rational $\overline{\mathcal {P}}$-Inner Functions

### Theorem 7.1

**(Fejér–Riesz theorem)** [27, Sect. 53] If $f(\lambda ) = \sum ^{n}_{i=-n}a_i\lambda ^i$ is a trigonometric polynomial of degree *n* such that $f(\lambda ) \ge 0$ for all $\lambda \in \mathbb {T}$, then there exists an analytic polynomial $D(\lambda ) = \sum ^n_{i=0}b_i\lambda ^i$ of degree *n* such that *D* is outer (that is, $D(\lambda )\ne 0$ for all $\lambda \in \mathbb {D}$) and

$$\begin{aligned} f(\lambda )=|D(\lambda )|^2 \end{aligned}$$

for all $$\lambda \in \mathbb {T}$$.

Recall that for every $a \ne 0$ in $H^\infty (\mathbb {D})$ there is an outer-inner factorization. Rational inner functions can be written in the form $c\prod \limits _{i=1}^{n}B_{\alpha _{i}}$ for some $n \ge 1$ and $\alpha _1, \dots , \alpha _n \in \mathbb {D}$ and $c \in \mathbb {C}$.

### Definition 7.2

[6, Definition 3.1] The degree deg(*h*) of a rational $\Gamma$-inner function *h* is defined to be $h_*(1)$, where $h_* : \mathbb {Z}=\pi _1(\mathbb {T}) \rightarrow \pi _1(b\Gamma )$ is the homomorphism of fundamental groups induced by *h* when it is regarded as a continuous map from $\mathbb {T}$ to $b\Gamma$.

Recall that, by [6, Proposition 3.3], for any rational $\Gamma$-inner function $h=(s, p)$, deg(*h*) is the degree deg(*p*) (in the usual sense) of the finite Blaschke product *p*.

### Definition 7.3

Let *g* be a polynomial of degree less than or equal to *n*, where $n \ge 0$. Then we define the polynomial $g^{\sim n}$ by

$$\begin{aligned} g^{\sim n}(\lambda )= \lambda ^n \overline{g(1/{\overline{\lambda }})}. \end{aligned}$$

### Definition 7.4

The degree of a rational $\overline{\mathcal {P}}$-inner function $x = (a, s, p)$ is defined to be the pair of numbers $(deg \;a, deg \;p)$. We say that deg $x \le (m, n)$ if deg $a \le m$ and deg $p \le n$.

The next theorem provides a description of the structure of rational penta-inner functions of prescribed degree.

### Theorem 7.5

Let $x=(a, s, p) : \mathbb {D} \rightarrow \overline{\mathcal {P}}$ be a rational penta-inner function of degree (*m*, *n*). Let $a \ne 0$ and let an inner-outer factorization of *a* be given by $a = a_{\textrm{in}} a_{\textrm{out}}$, where $a_{\textrm{in}}$ is an inner function and $a_{\textrm{out}}$ is an outer function. Then there exist polynomials *A*, *E*, *D* such that

1. $$deg(A), deg(E), deg(D) \le n$$,
2. $$E^{\sim n} = E$$,
3. $$D(\lambda ) \ne 0$$ on $$\overline{\mathbb {D}}$$,
4. $$|E(\lambda )| \le 2|D(\lambda )|$$ on $$\overline{\mathbb {D}}$$,
5. *A* is an outer polynomial such that $|A(\lambda )|^2 = |D(\lambda )|^2 - \frac{1}{4}|E(\lambda )|^2$ on $\mathbb {T}$,
6. $$a = a_{\textrm{in}} \dfrac{A}{D}$$ on $$\overline{\mathbb {D}}$$,
7. $$s = \dfrac{E}{D}$$ on $$\overline{\mathbb {D}}$$,
8. $$p = \dfrac{D^{\sim n}}{D}$$ on $$\overline{\mathbb {D}}$$.

### Proof

Suppose that $x=(a, s, p)$ is a rational penta-inner function. By Lemma 6.2, $h=(s, p)$ is a rational $\Gamma$-inner function. By [2, Corollary 6.10], *p* can be written in the form

$$\begin{aligned} p(\lambda ) = c\frac{\lambda ^kD^{\sim (n-k)}(\lambda )}{D(\lambda )}, \end{aligned}$$

where $|c|=1$, $0 \le k \le n$ and *D* is a polynomial of degree $n-k$ such that $D(0)=1$. Therefore, by [6, Proposition 2.2], there exist polynomials *E* and *D* such that

7.1 $$\begin{aligned} \text {(i)}&\text {deg}(E), \text {deg}(D) \le n, \nonumber \\ \text {(ii)}&E^{\sim n}=E,\nonumber \\ \text {(iii)}&D(\lambda ) \ne 0 \textit{ on } \overline{\mathbb {D}},\nonumber \\ \text {(iv)}&|E(\lambda )| \le 2 |D(\lambda )| \textit{ on } \overline{\mathbb {D}},\nonumber \\ \text {(v)}&s=\dfrac{E}{D} \textit{ on } \overline{\mathbb {D}},\nonumber \\ \text {(vi)}&p = \dfrac{D^{\sim n}}{D} \text { on } \overline{\mathbb {D}}. \end{aligned}$$

By assumption $x = (a, s, p)$ is a $\overline{\mathcal {P}}$-inner function, and so, for almost all $\lambda \in \mathbb {T}, \; (a(\lambda ), s(\lambda ), p(\lambda )) \in b\overline{\mathcal {P}}$, which implies

$$\begin{aligned} |a_{\textrm{out}}(\lambda )|^2 = 1-\frac{1}{4}|s(\lambda )|^2, \; \text { since } |a_{\textrm{in}}(\lambda )| = 1 \text { almost everywhere on } \mathbb {T}. \end{aligned}$$

Thus

$$\begin{aligned} |a_{\textrm{out}}(\lambda )|^2 = 1-\frac{1}{4}\frac{|E(\lambda )|^2}{|D(\lambda )|^2} \ \ \text { since } s(\lambda ) = \frac{E(\lambda )}{D(\lambda )}, \end{aligned}$$

and so,

7.2 $$\begin{aligned} |a_{\textrm{out}}(\lambda )|^2|D(\lambda )|^2 = |D(\lambda )|^2-\frac{1}{4}|E(\lambda )|^2. \end{aligned}$$

By [6, Proposition 2.2], $|E(\lambda )| \le 2|D(\lambda )|$. By the Fejér–Riesz Theorem, since $|D(\lambda )|^2-\frac{1}{4}|E(\lambda )|^2 \ge 0$, there exists an analytic polynomial *A* of degree $\le n$ such that *A* is outer and

7.3 $$\begin{aligned} |A(\lambda )|^2=|D(\lambda )|^2-\frac{1}{4}|E(\lambda )|^2 \end{aligned}$$

for all $$\lambda \in \mathbb {T}$$.

From Eqs. (7.2) and (7.3) we have, $|A(\lambda )|^2 = |a_{\textrm{out}}(\lambda )|^2 |D(\lambda )|^2$. Note that $D(\lambda )\ne 0$ on $\overline{\mathbb {D}}$. Thus $|a_{\textrm{out}}(\lambda )| = \left| \dfrac{A}{D}(\lambda )\right|$ for $\lambda \in \mathbb {T}$, and so $\dfrac{A}{D}$ is an outer function such that $|a(\lambda )| = \left| \dfrac{A}{D}(\lambda )\right|$ for almost all $\lambda \in \mathbb {T}$. Since outer factors are unique up to unimodular constant multiples, there exists $\omega \in \mathbb {T}$ such that

$$\begin{aligned} a_{\textrm{out}}(\lambda ) = \omega \dfrac{A(\lambda )}{D(\lambda )}. \end{aligned}$$

Therefore, $a = a_{\textrm{in}} \dfrac{A}{D}$ on $\overline{\mathbb {D}}$, after replacement of *A* by $\omega A$. $\square$

### Remark 7.6

Results similar to our Theorem 7.5 were announced on ArXiv in [25].

### Example 7.7

Consider a rational $\overline{\mathcal {P}}$-inner function $x(\lambda ) = (\lambda ^m, 0, \lambda )$ for $\lambda \in \mathbb {D}$. It is easy to see that polynomials described in Theorem 7.5 for this function are the following: $E(\lambda ) = 0$, $D(\lambda ) = 1$, $D^{\sim 1}(\lambda ) = \lambda$, $A(\lambda ) = 1$. Since $a(\lambda ) = \lambda ^m$ is inner, $a_{\textrm{in}}= a$ and so $a_{\textrm{in}}(\lambda ) = \lambda ^m$.

### Theorem 7.8

Let $h = (s, p) : \mathbb {D} \rightarrow \Gamma$ be a rational $\Gamma$-inner function of degree *n*. Let *E*, *D* be defined by equations (7.1) ( [6, Proposition 2.2]). Let *A* be an outer polynomial such that

7.4 $$\begin{aligned} |A(\lambda )|^2 = |D(\lambda )|^2-\frac{1}{4}|E(\lambda )|^2. \end{aligned}$$

Then, for every finite Blaschke product *B* and $|c|=1$, $x=\left( c B \dfrac{A}{D}, \dfrac{E}{D}, \dfrac{D^{\sim n}}{D}\right)$ is a rational $\overline{\mathcal {P}}$-inner function.

### Proof

Let *a*, *s*, *p* be defined by

$$\begin{aligned} a = c B \dfrac{A}{D}, \; \; \; s = \dfrac{E}{D} \; \; \; \text { and } p = \dfrac{D^{\sim n}}{D}. \end{aligned}$$

Let us show that $x = (a, s, p)$ is a rational $\overline{\mathcal {P}}$-inner function. We have to prove that $x : \mathbb {D}\rightarrow \overline{\mathcal {P}}$ and, for almost all $\lambda \in \mathbb {T}, \; x(\lambda ) \in b\overline{\mathcal {P}}$.

By assumption $h = (s, p) : \mathbb {D} \rightarrow \Gamma$ is a rational $\Gamma$-inner function, which means $|p(\lambda )| = 1, \ |s(\lambda )| \le 2 \ \text { and } \ (\overline{s}p)(\lambda ) = s(\lambda )$, for almost all $\lambda \in \mathbb {T}$. Now we need to show that for almost all $\lambda \in \mathbb {T}, \; |a(\lambda )| = \sqrt{1-\frac{1}{4}|s(\lambda )|^2}$. For almost all $\lambda \in \mathbb {T}$,

$$\begin{aligned} |a(\lambda )|^2&= \left| c B(\lambda ) \dfrac{A(\lambda )}{D(\lambda )}\right| ^2 = \dfrac{|A(\lambda )|^2}{|D(\lambda )|^2} \; \; \; \text { (since } |c| = 1 \text { and } |B(\lambda )| = 1 \text { on } \mathbb {T}) \\&= \dfrac{|D(\lambda )|^2-\frac{1}{4}|E(\lambda )|^2}{|D(\lambda )|^2} = 1-\frac{1}{4}\left| \frac{E(\lambda )}{D(\lambda )}\right| ^2 \\&= 1-\frac{1}{4}|s(\lambda )|^2. \end{aligned}$$

Let us show that $x = (a, s, p) = \left( c B \dfrac{A}{D}, \dfrac{E}{D}, \dfrac{D^{\sim n}}{D}\right)$ maps $\mathbb {D}$ to $\overline{\mathcal {P}}$, that is, $x(\lambda ) = (a(\lambda ), s(\lambda ), p(\lambda )) \in \overline{\mathcal {P}}$ for all $\lambda \in \mathbb {D}$. By the construction, $D(\lambda ) \ne 0$ on $\overline{\mathbb {D}}$, and so $(a(\lambda ), s(\lambda ), p(\lambda ))$ is analytic on $\mathbb {D}$. By Theorem 2.10, for each $\lambda \in \mathbb {D}, \; x(\lambda ) \in \overline{\mathcal {P}}$ if and only if $|\Psi _z(x(\lambda ))| \le 1 \text { for all } z \in \mathbb {D},$ where

$$\begin{aligned} \Psi _z(x(.)) :&\; \mathbb {D} \rightarrow \mathbb {C} \\&\; \lambda \mapsto (1-|z|^2) \; \frac{a(\lambda )}{1-s(\lambda )z+p(\lambda )z^2}. \end{aligned}$$

For all $\lambda \in \mathbb {D}$, $(s(\lambda ), p(\lambda )) \in \Gamma$, and so $1-s(\lambda )z+p(\lambda )z^2\ne 0$ for all $z \in \mathbb {D}$. Hence, for every $z \in \mathbb {D},\; \Psi _z(x(.))$ is analytic on $\mathbb {D}$. For fixed $z \in \mathbb {D}$, by the maximum principle, to prove that $|\Psi _z(x(\lambda ))| \le 1$ for all $\lambda \in \mathbb {D}$, it suffices to show that $|\Psi _z(x(\lambda ))| \le 1$ for all $\lambda \in \mathbb {T}$. We have shown above that, for almost all $\lambda \in \mathbb {T}, \; (a(\lambda ), s(\lambda ), p(\lambda )) \in b\overline{\mathcal {P}}$. Thus, for all $\lambda \in \mathbb {T}$, $|a(\lambda )| = \sqrt{1-\frac{1}{4}|s(\lambda )|^2}, \; |p(\lambda )| = 1, \; |s(\lambda )| \le 2$ and $s(\lambda ) = \overline{s(\lambda )}p(\lambda )$, and so $(s(\lambda ), p(\lambda )) = (\beta +{\overline{\beta }}p, p)(\lambda ) \in b\Gamma$, where $\beta (\lambda )=\frac{1}{2}s(\lambda )$. One can see that, for all $\lambda \in \mathbb {T}$,

$$\begin{aligned} \left| 1-\dfrac{\frac{1}{2}s(\lambda ){\overline{\beta }}(\lambda )}{1+\sqrt{1-|\beta (\lambda )|^2}}\right|&= \left| 1-\frac{\frac{1}{4}|s(\lambda )|^2}{1+\sqrt{1-\frac{1}{4}|s(\lambda )|^2}}\right| \\&=\left| \frac{1+\sqrt{1-\frac{1}{4}|s(\lambda )|^2}-\frac{1}{4}|s(\lambda )|^2}{1+\sqrt{1-\frac{1}{4}|s(\lambda )|^2}}\right| \\&= \left| \frac{\sqrt{1-\frac{1}{4}|s(\lambda )|^2} \left( 1 +\sqrt{1-\frac{1}{4}|s(\lambda )|^2} \right) }{1+\sqrt{1-\frac{1}{4}|s(\lambda )|^2}}\right| \\&= \sqrt{1-\frac{1}{4}|s(\lambda )|^2} = |a(\lambda )|. \end{aligned}$$

By Theorem 2.10 (3) $\Leftrightarrow$ (5), for each $\lambda \in \mathbb {T}$,

$$\begin{aligned} |a(\lambda )| \le \left| 1-\dfrac{\frac{1}{2}s(\lambda ){\overline{\beta }}(\lambda )}{1+\sqrt{1-|\beta (\lambda )|^2}}\right| \text { if and only if } |\Psi _z(a(\lambda ), s(\lambda ), p(\lambda ))| \le 1 \; \text {for all} \; z \in \mathbb {D}. \end{aligned}$$

Hence, by the maximum principle, for all $z, \lambda \in \mathbb {D}$, $|\Psi _z(a(\lambda ), s(\lambda ), p(\lambda ))| \le 1$. Thus, by Theorem 2.10, $x(\lambda ) = (a(\lambda ), s(\lambda ), p(\lambda )) \in \overline{\mathcal {P}}$ for all $\lambda \in \mathbb {D}$. $\square$

### Theorem 7.9

**(Converse to Theorem** **7.5****)** Suppose polynomials *A*, *E*, *D* satisfy

1. $$deg(A), deg(E), deg(D) \le n$$,
2. $$E^{\sim n} = E$$,
3. $$D(\lambda ) \ne 0$$ on $$\overline{\mathbb {D}}$$,
4. $$|E(\lambda )| \le 2|D(\lambda )|$$ on $$\overline{\mathbb {D}}$$,
5. *A* is an outer polynomial such that $|A(\lambda )|^2 = |D(\lambda )|^2-\frac{1}{4}|E(\lambda )|^2$ for $\lambda \in \mathbb {T}$,
6. $a_{\textrm{in}}$ is a rational inner function on $\mathbb {D}$ of degree $\le m$.

Let *a*, *s*, *p* be defined by

$$\begin{aligned} a = a_{\textrm{in}}\dfrac{A}{D}, \; \; \; s = \dfrac{E}{D} \; \; \; \text { and } \; \; \; p = \dfrac{D^{\sim n}}{D} \; \; \; \text { on } \overline{\mathbb {D}}. \end{aligned}$$

Then

$$\begin{aligned} x = \left( a_{\textrm{in}} \dfrac{A}{D}, \dfrac{E}{D},\dfrac{D^{\sim n}}{D}\right) \end{aligned}$$

is a rational $\overline{\mathcal {P}}$-inner function of degree less than or equal $(m+n, n)$.

### Proof

By the converse of [6, Proposition 2.2], $h = (s, p)$, where

$$\begin{aligned} s = \dfrac{E}{D} \; \; \; \text { and } \; \; \; p = \dfrac{D^{\sim n}}{D}, \end{aligned}$$

is a rational $\Gamma$-inner function of degree at most *n*. Since the rational inner functions on $\mathbb {D}$ are precisely the finite Blaschke products, the statement of the theorem follows from Theorem 7.8. $\square$

## Construction of Rational $\overline{\mathcal {P}}$-Inner Functions

In this section, we describe an algorithm for the construction of rational $\overline{\mathcal {P}}$-inner function from certain interpolation data. Firstly we recall some notions and statements from [6] which were useful for the construction of rational $\Gamma$-inner functions.

### Definition 8.1

[6, p. 140] Let $h =(s, p)$ be a rational $\Gamma$-inner function of degree *n*. Let *E* and *D* be as in Eq. (7.1) ([6, Proposition 2.2]). The royal polynomial $R_h$ of *h* is defined by

8.1 $$\begin{aligned} R_h(\lambda ) = 4D(\lambda )D^{\sim n}(\lambda )-E(\lambda )^2. \end{aligned}$$

We call the points $\lambda \in \overline{\mathbb {D}}$ such that $h(\lambda ) \in \mathcal {R}_{\Gamma }$ the *royal nodes* of *h* and, for such $\lambda$, we call $h(\lambda )$ a *royal point* of *h*, that is, $4p(\lambda )-s(\lambda )^2=0$. Since $D(\lambda )\ne 0$ on $\overline{\mathbb {D}}$, the royal nodes of *h* exactly correspond to the zeros of the royal polynomial $R_h$. Hence, $\lambda \in \overline{\mathbb {D}}$ is a royal node of *h* if and only if $R_h(\lambda ) = 0$.

### Definition 8.2

[6, Definition 3.4] We say that a polynomial *f* is *n*-symmetric if deg$(f)\le n$ and $f^{\sim n} = f$. For any set $E \subset \mathbb {C}$, ord$_E(f)$ will denote the number of zeros of *f* in *E*, counted with multiplicity, and ord$_0(f)$ will mean the same as ord$_{\{0\}}(f)$.

### Definition 8.3

[6, Definition 4.1] A non-zero polynomial *R* is *n*-balanced if deg$(R) \le 2n, \; R$ is 2*n*-symmetric and $\lambda ^{-n}R(\lambda ) \ge 0$ for all $\lambda \in \mathbb {T}$.

### Proposition 8.4

[6, Proposition 3.5] Let *h* be a rational $\Gamma$-inner function of degree *n* and let $R_h$ be the royal polynomial of *h* as defined by Eq. (8.1). Then $R_h$ is 2*n*-symmetric and the zeros of $R_h$ that lie on $\mathbb {T}$ have either even or infinite order.

### Definition 8.5

[6, Definition 3.6] Let *h* be a rational $\Gamma$-inner function such that $h(\overline{\mathbb {D}})\nsubseteq \mathcal {R}_\Gamma \cap \Gamma$ and let $R_h$ be the royal polynomial of *h*. If $\sigma$ is a zero of $R_h$ of order $\ell$, we define the multiplicity $\#\sigma$ of $\sigma$ (as a royal node of *h*) by

$$\begin{aligned} \#\sigma = {\left\{ \begin{array}{ll} \ell &{} \text { if } \sigma \in \mathbb {D} \\ \frac{1}{2}\ell &{} \text { if } \sigma \in \mathbb {T}. \end{array}\right. } \end{aligned}$$

We next present a description of rational penta-inner functions (*a*, *s*, *p*) in terms of the zeros of *a*, *s* and $s^2 -4p$.

### Theorem 8.6

Suppose that $\alpha _1, \alpha _2, \dots , \alpha _{k_0} \in \mathbb {D}$ and $\eta _1, \eta _2, \dots , \eta _{k_1} \in \mathbb {T}$, where $2k_0+k_1=n$ and suppose that $\beta _1, \beta _2, \dots , \beta _m \in \mathbb {D}$. Suppose that $\sigma _1, \dots , \sigma _n$ in $\overline{\mathbb {D}}$ are distinct from $\eta _1, \dots , \eta _{k_1}$. Then there exists a rational $\overline{\mathcal {P}}$-inner function $x = (a, s, p)$ of degree less than or equal $(m+n, n)$ such that

1. the zeros of *a* in $\mathbb {D}$, repeated according to multiplicity, are $\beta _1, \beta _2, \dots , \beta _m$,
2. the zeros of *s* in $\overline{\mathbb {D}}$, repeated according to multiplicity, are $\alpha _1, \alpha _2, \dots , \alpha _{k_0}$ and $\eta _1, \eta _2, \dots , \eta _{k_1}$,
3. the royal nodes of (*s*, *p*) are $\sigma _1, \dots , \sigma _n$.

Such a function *x* can be constructed as follows. Let $t_+ \; \>\; 0$ and let $t \in \mathbb {R} \setminus \{0\}$. Let *R* and *E* be defined by

$$\begin{aligned} R(\lambda ) = t_{+} \prod _{j=1}^{n} (\lambda -\sigma _j)(1-\overline{\sigma _j}\lambda ), \\ E(\lambda ) = t\prod _{j=1}^{k_0} (\lambda -\alpha _j)(1-\overline{\alpha _j}\lambda ) \prod _{j=1}^{k_1} ie ^{-i \theta _j/2}(\lambda -\eta _j), \end{aligned}$$

where $\eta _j = e ^{i\theta _j}, \; 0 \le \theta _j \; <\; 2\pi$. Let $a_{\textrm{in}} : \mathbb {D} \rightarrow \overline{\mathbb {D}}$ be defined by

8.2 $$\begin{aligned} a_{\textrm{in}}(\lambda ) = c\prod _{i=1}^{m} B_{\beta _i}(\lambda ), \end{aligned}$$

where $$|c|=1$$ and $$\beta _i \in \mathbb {D}, \; i=1, \dots , m$$.

(i) There exist outer polynomials *D* and *A* of degree at most *n* such that
  8.3 $$\begin{aligned} \lambda ^{-n}R(\lambda )+|E(\lambda )|^2 = 4|D(\lambda )|^2 \end{aligned}$$
  and
  8.4 $$\begin{aligned} \lambda ^{-n}R(\lambda ) = 4|A(\lambda )|^2 \end{aligned}$$
  for all $$\lambda \in \mathbb {T}$$.
(ii) The function *x* defined by
  8.5 $$\begin{aligned} x=(a, s, p)=\Big (a_{\textrm{in}}\frac{A}{D}, \frac{E}{D}, \frac{D^{\sim n}}{D}\Big ) \end{aligned}$$
  is a rational $\overline{\mathcal {P}}$-inner function such that deg$(x) \le (m+n, n)$ and conditions (1), (2) and (3) hold. The royal polynomial of (*s*, *p*) is *R*.

### Proof

(i) By [6, Lemma 4.4], *R* is *n*-balanced, and so $\lambda ^{-n}R(\lambda ) \ge 0$ for all $\lambda \in \mathbb {T}$. Therefore,
  $$\begin{aligned} \lambda ^{-n}R(\lambda )+|E(\lambda )|^2 \ge 0 \text { for all } \lambda \in \mathbb {T}. \end{aligned}$$
  By the Fejér–Riesz theorem, there exist outer polynomials *A* and *D* of degree at most *n* such that
  $$\begin{aligned} \lambda ^{-n}R(\lambda ) = 4|A(\lambda )|^2 \; \text { for all } \lambda \in \mathbb {T} \end{aligned}$$
  and
  $$\begin{aligned} \lambda ^{-n}R(\lambda )+|E(\lambda )|^2 = 4|D(\lambda )|^2 \; \text { for all } \lambda \in \mathbb {T}. \end{aligned}$$
(ii) By [6, Theorem 4.8], the function *h* defined by
  $$\begin{aligned} h=(s, p)=\Big (\frac{E}{D}, \frac{D^{\sim n}}{D}\Big ) \end{aligned}$$
  is a rational $\Gamma$-inner function such that deg$(h)=n$ and conditions (2) and (3) hold. The royal polynomial of *h* is *R*. By Eqs. (8.3) and (8.4),
  $$\begin{aligned} |A(\lambda )|^2= & {} |D(\lambda )|^2-\frac{1}{4}|E(\lambda )|^2. \end{aligned}$$
  Therefore, by Proposition 7.8,
  $$\begin{aligned} x = \Big (a_{\textrm{in}}\frac{A}{D}, \frac{E}{D}, \frac{D^{\sim n}}{D}\Big ) \end{aligned}$$
  is a rational $\overline{\mathcal {P}}$- inner function. By the definition (8.2) of $a_{\textrm{in}}$, the zeros of $a_{\textrm{in}}$ are $\beta _1, \dots , \beta _m$, while, since *A* is an outer polynomial, *A* has no zeros in $\mathbb {D}$. Hence the zeros of $a = a_{\textrm{in}}\dfrac{A}{D}$ in $\mathbb {D}$ are $\beta _1, \dots , \beta _m$, as required for (1).

$$\square $$

### Theorem 8.7

Let $x=(a, s, p)$ be a rational $\overline{\mathcal {P}}$-inner function of degree $(m+n, n)$ such that

1. the zeros of *a*, repeated according to multiplicity, are $\beta _1, \beta _2, \dots , \beta _m \in \mathbb {D}$,
2. the zeros of *s*, repeated according to multiplicity, are $\alpha _1, \alpha _2, \dots , \alpha _{k_0} \in \mathbb {D}$ and $\eta _1, \eta _2, \dots , \eta _{k_1} \in \mathbb {T}$, where $2k_0+k_1=n$,
3. the royal nodes of (*s*, *p*) are $\sigma _1, \dots , \sigma _n \in \overline{\mathbb {D}}$.

There exists some choice of $c \in \mathbb {T}, \; t_{+} \; \>\; 0, \; t \in \mathbb {R} \setminus \{0\}$ and $\omega \in \mathbb {T}$ such that the recipe in Theorem 8.6 with these choices produces the function *x*.

### Proof

By Lemma 6.2, $h=(s, p)$ is a rational $\Gamma$-inner function of degree *n*. As in [6, Proposition 4.9], there exists some choice of $t_{+} \; \>\; 0, \; t \in \mathbb {R} \setminus \{0\}$ and $\omega \in \mathbb {T}$ such that the recipe of [6, Theorem 4.8] produces the function *h*. Let us give those steps.

By [6, Proposition 2.2], there exist polynomials $E_1$ and $D_1$ such that deg$(E_1)$, deg$(D_1) \le n$, $E_1$ is *n*-symmetric, $D_1(\lambda ) \ne 0$ on $\overline{\mathbb {D}}$, and

$$\begin{aligned} s = \dfrac{E_1}{D_1} \; \text { and } \; \; p = \dfrac{D_{1}^{\sim n}}{D_1} \text { on } \overline{\mathbb {D}}. \end{aligned}$$

By hypothesis, the zeros of *s*, repeated according to multiplicity, are $\alpha _1, \alpha _2, \dots , \alpha _{k_0}$ and $\eta _1, \eta _2, \dots , \eta _{k_1}$, where $2k_0+k_1=n$. Since $E_1$ is *n*-symmetric, by [6, Lemma 4.6], there exists $t \in \mathbb {R} \setminus \{0\}$ such that

$$\begin{aligned} E_1(\lambda ) = t\prod _{j=1}^{k_0} (\lambda -\alpha _j)(1-\overline{\alpha _j}\lambda ) \prod _{j=1}^{k_1} ie ^{-i \theta _j/2}(\lambda -\eta _j), \end{aligned}$$

where $\eta _j = e^{i \theta _j}$ for $j =1, \dots , k_1.$ The royal nodes of *h* are assumed to be $\sigma _1, \dots , \sigma _n$. By [6, Proposition 4.5], for the royal polynomial $R_1$ of *h*, there exists $t_{+} \; \>\; 0$ such that

$$\begin{aligned} R_1(\lambda ) = t_{+} \prod _{j=1}^{n} Q_{\sigma _j}(\lambda ), \end{aligned}$$

where $$Q_{\sigma _j}(\lambda )= (\lambda -\sigma _j)(1-\overline{\sigma _j}\lambda )$$, $$j= 1, \dots ,n$$.

Since $E_1$ and $R_1$ coincide with *E* and *R* in the construction of Theorem 8.6, for a suitable choice of $t_{+} \; \>\; 0$ and $t \in \mathbb {R} \setminus \{0\}$, $D_1$ is a permissible choice for $\omega D$ for some $\omega \in \mathbb {T}$, as a solution of Eq. (8.3). By assumption the zeros of *a*, repeated according to multiplicity, are $\beta _1, \beta _2, \dots , \beta _m \in \mathbb {D}$. Then the inner part of *a* will be equal to $a_{\textrm{in}}^{1} = c_1 \prod \limits _{i=1}^{m}B_{\beta _i}$, where $|c_1|=1$. For the outer part of *a* there is an outer polynomial $A_1$ such that

$$\begin{aligned} |A_1(\lambda )|^2= & {} |D_1(\lambda )|^2-\frac{1}{4}|E_1(\lambda )|^2 \\= & {} |D(\lambda )|^2-\frac{1}{4}|E(\lambda )|^2 \\= & {} \lambda ^{-n}R(\lambda ), \end{aligned}$$

for $\lambda \in \mathbb {T}$. By Eq. (8.4), $A_1 = c_2 A$ up to a constant $c_2$ such that $|c_2|=1$. Also, $a_{\textrm{in}}^{1}$ coincides with $a_{\textrm{in}}$ for a suitable choice of $c \in \mathbb {T}$. Hence the construction of Theorem 8.6 yields $x=(a, s, p)$ for the appropriate choices of $t_{+} \; \>\; 0$, $t \in \mathbb {R} \setminus \{0\}$, $\omega$ and $c \in \mathbb {T}$. $\square$

## A Special Case of Schwarz Lemma for $\mathcal {P}$

The classical Schwarz lemma gives a solvability criterion for a two-point interpolation problem in $\mathbb {D}$. In [4] a simple analogue of Schwarz lemma the for two-point $\mu$-synthesis was given. We consider a general linear subspace *E* of $\mathbb {C}^{n\times m}$ and the corresponding $\mu _E$ on $\mathbb {C}^{m\times n}$, as in Eq. (1.2). We shall denote by *N* the Nevanlinna class of functions on the disc [28] and if *F* is a matrix function on $\mathbb {D}$ then we write $F\in N$ to mean that each entry of *F* belongs to *N*. It then follows from Fatou’s Theorem that if $F\in N$ is an $m\times n$-matrix-valued function then

$$\begin{aligned} \lim _{r\rightarrow 1-} F(r\lambda ) \text{ exists } \text{ for } \text{ almost } \text{ all } \lambda \in \mathbb {T}. \end{aligned}$$

The following Schwarz lemma was proved in [4, Proposition 10.3].

### Proposition 9.1

Let $\lambda _0 \in \mathbb {D}\setminus \{0 \}$, let $W \in \mathbb {C}^{m\times n}$ and let *E* be a subset of $\mathbb {C}^{n\times m}$. There exists $F \in N\cap {{\,\textrm{Hol}\,}}(\mathbb {D},\mathbb {C}^{m\times n})$ such that

1. $$F(0) =0$$ and $$F(\lambda _0)= W$$,
2. $$\mu _E(F(\lambda )) < 1 $$ for all $$\lambda \in \mathbb {D}$$

if and only if $\mu _E(W) \le |\lambda _0|$.

In this section, we consider a simple case of a Schwarz lemma for the pentablock. We will need the following elementary technical lemma.

### Lemma 9.2

Let $$A = $$ $$\begin{bmatrix} \lambda _1 &{} 0 \\ a &{} \lambda _2 \end{bmatrix}$$, where $$\lambda _1, \; \lambda _2, \; a \in \mathbb {C}$$. Then the following conditions are equivalent:

(i) $$\lambda _1, \lambda _2 \in \overline{\mathbb {D}}, \; |a| \le (1 - |\lambda _1|^2)^{\frac{1}{2}}(1 - |\lambda _2|^2)^{\frac{1}{2}}$$,
(ii) $$\Vert A\Vert \le 1$$,
(iii) $$1-A^*A \ge 0$$.

### Definition 9.3

$H^{\infty }(\mathbb {D}, \mathbb {C}^{2\times 2})$ denotes the space of bounded analytic $2\times 2$ matrix-valued functions on $\mathbb {D}$ with the supremum norm:

$$\begin{aligned} \Vert f\Vert _{H^\infty } = \sup \limits _{z \in \mathbb {D}}\Vert f(z)\Vert _{\mathbb {C}^{2\times 2}}. \end{aligned}$$

### Definition 9.4

$L^{\infty }(\mathbb {T}, \mathbb {C}^{2\times 2})$ denotes the space of essentially bounded Lebesgue-measurable $2\times 2$ matrix-valued functions on $\mathbb {T}$ with the essential supremum norm:

$$\begin{aligned} \Vert f\Vert _{L^\infty } = ess \sup \limits _{|z|=1}\Vert f(z)\Vert _{\mathbb {C}^{2\times 2}}. \end{aligned}$$

### Lemma 9.5

If $g \in H^{\infty }(\mathbb {D}, \mathbb {C}^{2\times 2})$ and $\lambda _0 \in \mathbb {D}$ then $\Vert g(\lambda _0)\Vert _{\mathbb {C}^{2\times 2}} \le \Vert g\Vert _{L^\infty }$.

### Proof

Consider any unit vectors $x, y \in \mathbb {C}^{2}$ and the scalar function

$$\begin{aligned} f&:&\mathbb {D} \rightarrow \mathbb {C} \\&:&\lambda \longmapsto \langle g(\lambda )x, y \rangle _{\mathbb {C}^2}. \end{aligned}$$

Note that, for every $\lambda \in \mathbb {D}$, since $\Vert x\Vert _{\mathbb {C}^2} = \Vert y\Vert _{\mathbb {C}^2} = 1$

$$\begin{aligned} |f(\lambda )| = |\left\langle g(\lambda )x, y \right\rangle _{\mathbb {C}^2}|\le & {} \Vert g(\lambda )x\Vert _{\mathbb {C}^2} \; \Vert y\Vert _{\mathbb {C}^2} \; \; \; \; \; \text {(Cauchy--Schwarz inequality)}\\\le & {} \Vert g(\lambda )\Vert _{\mathbb {C}^{2\times 2}} \; \Vert x\Vert _{\mathbb {C}^2} \; \Vert y\Vert _{\mathbb {C}^2} \\\le & {} \Vert g\Vert _{H^\infty }. \end{aligned}$$

Thus *f* is bounded on $\mathbb {D}$. Since *g* is analytic on $\mathbb {D}$, it is easy to show that *f* is analytic on $\mathbb {D}$ and, for every $z_0 \in \mathbb {D}, \; f'(z_0) = \langle g'(z_0)x, y \rangle _{\mathbb {C}^2}$. By the maximum principle for scalar analytic functions, for every $\lambda _0 \in \mathbb {D}, \; |f(\lambda _0)| \le ess \sup \limits _{z \in \mathbb {T}} |f(z)|$, and so

$$\begin{aligned} \left| \left\langle g(\lambda _0)x, y \right\rangle _{\mathbb {C}^2}\right|\le & {} ess \sup \limits _{z \in \mathbb {T}} \left| \left\langle g(z)x, y \right\rangle \right| \\\le & {} ess \sup \limits _{z \in \mathbb {T}} \Vert g(z)\Vert _{\mathbb {C}^{2\times 2}} = \Vert g\Vert _{L^{\infty }}. \end{aligned}$$

Take the supremum of both sides in this inequality over unit vectors *x*, *y* to get

$$\begin{aligned} \Vert g(\lambda _0)\Vert _{\mathbb {C}^{2\times 2}} \le \Vert g\Vert _{L^{\infty }}. \end{aligned}$$

$$\square $$

The following statement is known and follows easily from Lemma 9.5.

### Corollary 9.6

If $F \in H^{\infty }(\mathbb {D}, \mathbb {C}^{2\times 2})$ and $F(0) = 0$ then, for any $\lambda _0 \in \mathbb {D}$,

$$\begin{aligned} \Vert F(\lambda _0)\Vert _{\mathbb {C}^{2\times 2}} \le |\lambda _0|\;\Vert F\Vert _{H^{\infty }}. \end{aligned}$$

We next describe a special case of a Schwarz lemma for the pentablock.

### Theorem 9.7

Let $\lambda _0 \in \mathbb {D} \setminus \{0\}$, and $(a_0, s_0, p_0) \in \overline{\mathcal {P}}$, where $s_0 = \lambda _1+\lambda _2, \; p_0 = \lambda _1\lambda _2$, for some $\lambda _1, \lambda _2 \in \mathbb {D}$. Then the following conditions are equivalent:

(i) $$|\lambda _1| \le |\lambda _0|, \; |\lambda _2| \le |\lambda _0|$$, and
  9.1 $$\begin{aligned} |a_0| \le |\lambda _0|\left( 1-\left| \dfrac{\lambda _1}{\lambda _0}\right| ^2\right) ^{\frac{1}{2}} \left( 1-\left| \dfrac{\lambda _2}{\lambda _0}\right| ^2\right) ^{\frac{1}{2}}. \end{aligned}$$
(ii) There exists an analytic map $F : \mathbb {D} \rightarrow \overline{\mathbb {B}^{2\times 2}}$ such that
  $$\begin{aligned} F(0) = 0 \text { and } F(\lambda _0) = \begin{bmatrix} \lambda _1 &{} 0 \\ a_0 &{} \lambda _2 \end{bmatrix}. \end{aligned}$$
  Furthermore, if (i) holds and $x = \pi \circ F$, then *x* is an analytic map from $\mathbb {D}$ to $\overline{\mathcal {P}}$ such that $x(0) = (0,0,0)$ and $x(\lambda _0) = (a_0, s_0, p_0)$.

### Proof

(i) $\Rightarrow$ (ii) By assumption, $|\lambda _1| \le |\lambda _0|, \; |\lambda _2| \le |\lambda _0|$ and

$$\begin{aligned} |a_0| \le |\lambda _0|\left( 1-\left| \dfrac{\lambda _1}{\lambda _0}\right| ^2\right) ^{\frac{1}{2}} \left( 1-\left| \dfrac{\lambda _2}{\lambda _0}\right| ^2\right) ^{\frac{1}{2}}. \end{aligned}$$

Define

9.2 $$\begin{aligned} F(\lambda ) = \frac{\lambda }{\lambda _0}\begin{bmatrix} \lambda _1 &{} 0 \\ a_0 &{} \lambda _2 \end{bmatrix} = \lambda \begin{bmatrix} \lambda _1 / \lambda _0 &{} 0 \\ a_0 / \lambda _0 &{} \lambda _2 / \lambda _0 \end{bmatrix}. \end{aligned}$$

By Lemma 9.2,

$$\begin{aligned} \left\| \begin{bmatrix} \dfrac{\lambda _1}{\lambda _0} &{} 0 \\ \dfrac{a_0}{\lambda _0} &{} \dfrac{\lambda _2}{\lambda _0} \end{bmatrix}\right\| _{\mathbb {C}^{2\times 2}} \le 1. \end{aligned}$$

Hence $\Vert F(\lambda )\Vert \le |\lambda |$ for all $\lambda \in \mathbb {D}$, and so $\Vert F\Vert _{\infty } \le 1$. From the definition (9.2) of *F*, we have

$$\begin{aligned} F(0) = \begin{bmatrix} 0 &{} 0 \\ 0 &{} 0 \end{bmatrix} \text { and } F(\lambda _0) = \begin{bmatrix} \lambda _1 &{} 0 \\ a_0 &{} \lambda _2 \end{bmatrix}. \end{aligned}$$

(ii) $\Rightarrow$ (i) Suppose (ii) is satisfied. By Corollary 9.6,

$$\begin{aligned} \left\| \begin{bmatrix} \lambda _1 &{} 0 \\ a_0 &{} \lambda _2 \end{bmatrix}\right\| _{\mathbb {C}^{2\times 2}} = \Vert F(\lambda _0)\Vert _{\mathbb {C}^{2\times 2}} \le |\lambda _0| \; \Vert F\Vert _{H^\infty }. \end{aligned}$$

By assumption $\Vert F\Vert _{H^\infty } \le 1$, and so $$\left\| \begin{bmatrix} \lambda _1 &{} 0 \\ a_0 &{} \lambda _2 \end{bmatrix}\right\| _{\mathbb {C}^{2\times 2}} \le |\lambda _0|$$, hence, $$\left\| \begin{bmatrix} \dfrac{\lambda _1}{\lambda _0} &{} 0 \\ \dfrac{a_0}{\lambda _0} &{} \dfrac{\lambda _2}{\lambda _0} \end{bmatrix}\right\| _{\mathbb {C}^{2\times 2}} \le 1. \; $$ By Lemma 9.2,

$$\begin{aligned}&\left\| \begin{bmatrix} \dfrac{\lambda _1}{\lambda _0} &{} 0 \\ \dfrac{a_0}{\lambda _0} &{} \dfrac{\lambda _2}{\lambda _0} \end{bmatrix}\right\| _{\mathbb {C}^{2\times 2}} \le 1 \text { if and only if } \\&\quad |a_0| \le |\lambda _0|\left( 1-\left| \dfrac{\lambda _1}{\lambda _0}\right| ^2\right) ^{\frac{1}{2}} \left( 1-\left| \dfrac{\lambda _2}{\lambda _0}\right| ^2\right) ^{\frac{1}{2}}, \; \; |\lambda _1| \le |\lambda _0| \text { and } |\lambda _2| \le |\lambda _0|. \end{aligned}$$

Let us consider $x = \pi \circ F$ on $\mathbb {D}$. By assumption $(a_0, s_0, p_0) \in \overline{\mathcal {P}}$. By Theorem 2.10 (6), since $\Vert F(\lambda )\Vert \le 1$ for each $\lambda \in \mathbb {D}$, $x(\lambda ) = \pi (F(\lambda )) = \dfrac{\lambda }{\lambda _0}(a_0, s_0, p_0)$ maps $\mathbb {D}$ to $\overline{\mathcal {P}}$. Therefore, $x : \mathbb {D} \rightarrow \overline{\mathcal {P}}$ is analytic on $\mathbb {D}$ and maps 0 to (0, 0, 0) and $\lambda _0$ to $(a_0, s_0, p_0)$. $\square$

## A Schwarz Lemma for the Symmetrized Bidisc $\Gamma$

In [11], Agler and Young proved the following theorems.

### Theorem 10.1

[11, Theorem 1.1] Let $\lambda _0 \in \mathbb {D}$ and $(s_0, p_0) \in \Gamma$. The following conditions are equivalent:

1. There exists an analytic function $\varphi : \mathbb {D} \rightarrow \Gamma$ such that $\varphi (0) = (0, 0)$ and $\varphi (\lambda _0) = (s_0, p_0)$;
2. $$|s_0| \; <\; 2$$ and
  $$\begin{aligned} \dfrac{2|s_0 - p_0\overline{s_0}| + |s_{0}^{2} - 4p_0|}{4 - |s_0|^2} \le |\lambda _0|; \end{aligned}$$
3. 10.1 $$\begin{aligned} \big | |\lambda _0|^2s_0 - p_0\overline{s_0}\big | + |p_0|^2 + (1 - |\lambda _0|^2) \dfrac{|s_0|^2}{4} - |\lambda _0|^2 \le 0; \end{aligned}$$
4. $$\begin{aligned} |s_0| \le \frac{2}{1 - |\lambda _0|^2}(|\lambda _0| |1 - p_0{\overline{\omega }}^2| - \big | |\lambda _0|^2 - p_0 {\overline{\omega }}^2\big |), \end{aligned}$$
  where $\omega$ is a complex number of unit modulus such that $s_0 = |s_0|\omega$.

Moreover, for any analytic function $\varphi = (\varphi _1, \varphi _2) : \mathbb {D} \rightarrow \Gamma$ such that $\varphi (0) = (0, 0)$,

$$\begin{aligned} \frac{1}{2}|\varphi _{1}^{'}(0)| + |\varphi _{2}^{'}(0)| \le 1. \end{aligned}$$

The following theorem shows the construction of an interpolating function $\varphi$ satisfying the inequalities of Theorem 10.1 with equality.

### Theorem 10.2

[11, Theorem 1.4] Let $\lambda _0 \in \mathbb {D}$, and $(s_0, p_0) \in \Gamma$ be such that $\lambda _0 \ne 0$, $|s_0| <2$ and

$$\begin{aligned} \dfrac{2|s_0 - p_0\overline{s}_0| + |s_{0}^{2} - 4p_0|}{4 - |s_0|^2} = |\lambda _0|. \end{aligned}$$

Then there exists an analytic function $\varphi : \mathbb {D} \rightarrow \Gamma$ such that $\varphi (0) = (0, 0)$ and $\varphi (\lambda _0) = (s_0, p_0)$, given explicitly as follows:

If $$|p_0| = |\lambda _0|$$, then

10.2 $$\begin{aligned} \varphi (\lambda ) = (0, \omega \lambda ), \end{aligned}$$

where $\omega$ is a complex number of unit modulus such that $\omega \lambda _0 = p_0$.

If $|p_0| \; <\; |\lambda _0|$, then $\varphi = (\varphi _1, \varphi _2)$ where

10.3 $$\begin{aligned} \varphi _1(\lambda )= & {} \frac{c \zeta \lambda }{(1 - {\overline{\lambda }}_{0}\lambda ) (1 + \overline{p}_1 \zeta ^2 v(\lambda ))}, \end{aligned}$$

10.4 $$\begin{aligned} v(\lambda )= & {} \frac{\lambda - \lambda _0}{1 - {\overline{\lambda }}_0 \lambda }, \; \; \; \; \; \zeta \lambda _0|s_0| = |\lambda _0| s_0, \; \; \; \; \; |\zeta | = 1,\nonumber \\ p_1= & {} \frac{p_0}{\lambda _0}, \; \; \; \; \; c = \frac{2}{|\lambda _0|}\{|{\overline{\lambda }}_0 - \overline{p}_0 \lambda _0 \zeta ^2| - |\lambda _0^2 \zeta ^2 - p_0|\},\nonumber \\ \varphi _2(\lambda )= & {} \frac{\lambda (\zeta ^2 v(\lambda ) + p_1)}{1 + \overline{p}_1 \zeta ^2 v(\lambda )}. \end{aligned}$$

### Lemma 10.3

Consider the rational function $\varphi = (\varphi _1, \varphi _2): \mathbb {D} \rightarrow \Gamma$, where $\varphi _1, \varphi _2$ are defined as in Eqs. (10.3) and (10.4) above. Define the polynomials *E* and *D* by the equations:

$$\begin{aligned} E(\lambda )= & {} c \lambda ,\\ D(\lambda )= & {} {\overline{\zeta }}\{(1-\overline{\lambda _0}\lambda ) + \overline{p_1}\zeta ^2 (\lambda - \lambda _0)\}, \end{aligned}$$

where

$$\begin{aligned} |\zeta | = 1, \; \; \; p_1 = \dfrac{p_0}{\lambda _0}, \; \; \; \; \; c = \dfrac{2}{|\lambda _0|}\{|{\overline{\lambda }}_0 - \overline{p}_0 \lambda _0 \zeta ^2| - |\lambda _0^2 \zeta ^2 - p_0|\}. \end{aligned}$$

Then $\varphi _1 = \dfrac{E}{D}$ and $\varphi _2 = \dfrac{D^{\sim 2}}{D}$. Moreover, $E^{\sim 2} = E$ and $|E(\lambda )| \le 2 |D(\lambda )|$ on $\overline{\mathbb {D}}$.

### Proof

Let us check that $\varphi _1(\lambda ) = \dfrac{E(\lambda )}{D(\lambda )}$.

$$\begin{aligned} \frac{E(\lambda )}{D(\lambda )}= & {} \frac{c\lambda }{{\overline{\zeta }}\{(1-\overline{\lambda _0}\lambda ) + \overline{p_1}\zeta ^2 (\lambda - \lambda _0)\}} \times \frac{\zeta }{\zeta } \\= & {} \frac{c \zeta \lambda }{(1-\overline{\lambda _0}\lambda ) + \overline{p_1}\zeta ^2 (\lambda - \lambda _0)} \\= & {} \frac{c \zeta \lambda }{(1-\overline{\lambda _0}\lambda ) (1 + \overline{p_1}\zeta ^2 v(\lambda ))} = \varphi _1(\lambda ). \end{aligned}$$

To check that $\varphi _2(\lambda ) = \dfrac{D^{\sim 2}(\lambda )}{D(\lambda )}$, we need to find $D^{\sim 2}(\lambda )$.

$$\begin{aligned} D^{\sim 2}(\lambda ) = \lambda ^2 \overline{D(1 / {\overline{\lambda }})}= & {} \lambda ^2 \overline{{\overline{\zeta }}\left\{ \left( 1- \frac{\overline{\lambda _0}}{{\overline{\lambda }}}\right) + \overline{p_1}\zeta ^2\left( \frac{\ 1 \ }{\ {\overline{\lambda }} \ }-\lambda _0\right) \right\} } \\= & {} \lambda ^2 \zeta \left\{ \left( 1- \frac{\lambda _0}{\lambda }\right) + p_1 {\overline{\zeta }}^2 \left( \frac{1}{\lambda } - \overline{\lambda _0}\right) \right\} \\= & {} \lambda \zeta (\lambda - \lambda _0) + \lambda p_1 {\overline{\zeta }}(1-\overline{\lambda _0}\lambda ). \end{aligned}$$

Now,

$$\begin{aligned} \frac{D^{\sim 2}(\lambda )}{D(\lambda )}= & {} \frac{\lambda \zeta (\lambda - \lambda _0) + \lambda p_1 {\overline{\zeta }}(1-\overline{\lambda _0}\lambda )}{{\overline{\zeta }}\{(1-\overline{\lambda _0}\lambda ) + \overline{p_1}\zeta ^2 (\lambda - \lambda _0)\}} \times \frac{\left( \dfrac{\zeta }{1-\overline{\lambda _0}\lambda }\right) }{\left( \dfrac{\zeta }{1-\overline{\lambda _0}\lambda }\right) } \\= & {} \frac{\lambda \zeta ^2 \left( \dfrac{\lambda - \lambda _0}{1-\overline{\lambda _0}\lambda }\right) + \lambda p_1}{1+\overline{p_1}\zeta ^2 \left( \dfrac{\lambda - \lambda _0}{1-\overline{\lambda _0}\lambda }\right) } \\= & {} \frac{\lambda (\zeta ^2 v(\lambda )+ p_1)}{1+\overline{p_1} \zeta ^2 v(\lambda )} = \varphi _2(\lambda ), \end{aligned}$$

where $$v(\lambda ) = \dfrac{\lambda - \lambda _0}{1-\overline{\lambda _0}\lambda }$$.

We would like to show that $E^{\sim 2} = E$. For $\lambda \in \mathbb {D}$,

$$\begin{aligned} E^{\sim 2}(\lambda ) = \lambda ^2 \overline{E(1/{\overline{\lambda }})}= & {} \lambda ^2 \overline{\left( c \; \frac{\ 1 \ }{\ {\overline{\lambda }} \ }\right) } \\= & {} c \; \lambda = E(\lambda ), \;\; \text {since} \; c \in \mathbb {R}. \end{aligned}$$

By assumption, $\varphi = (\varphi _1, \varphi _2): \mathbb {D} \rightarrow \Gamma$, and we have proved that $\varphi _1 = \dfrac{E}{D}$, thus $|E(\lambda )| \le 2 |D(\lambda )|$ on $\overline{\mathbb {D}}$. $\square$

### Proposition 10.4

Let $h = (s, p)$ be the function from $\mathbb {D}$ to $\Gamma$ defined by

$$\begin{aligned} s(\lambda ) = \varphi _1(\lambda ), \; p(\lambda ) = \varphi _2(\lambda ), \; \lambda \in \mathbb {D}, \end{aligned}$$

as in Eqs. (10.3) and (10.4). Then *h* is a rational $\Gamma$-inner function of degree 2.

### Proof

By Lemma 10.3 and by the converse part of [6, Proposition 2.2], *h* is a rational $\Gamma$-inner function.

Another way to prove that *h* is a rational $\Gamma$-inner function is as follows. One can easily see that $h = (s, p)$ is a rational function, and so there are only finitely many singularities of this function. Hence we can extend *h* continuously to almost all points in $\mathbb {T}$.

Let us show that for almost all $\lambda \in \mathbb {T}, \; h(\lambda ) \in b\Gamma$. We need to show that, for almost all $\lambda \in \mathbb {T}, \; |p(\lambda )| = 1, \; |s(\lambda )| \le 2$ and $s(\lambda ) = \overline{s(\lambda )} p(\lambda )$. Since $v(\lambda ) = \dfrac{\lambda -\lambda _0}{1-\overline{\lambda _0}\lambda }$ is an inner function from $\mathbb {D}$ to $\overline{\mathbb {D}}$, for almost all $\lambda \in \mathbb {T}, \; |v(\lambda )| = 1$.

$$\begin{aligned} |p(\lambda )|= & {} \frac{|\lambda | |\zeta ^2 v(\lambda ) + p_1|}{|1 + \overline{p_1} \zeta ^2 v(\lambda )|} = \frac{|\zeta ^2 v(\lambda ) + p_1|}{|\zeta ^2 \overline{\zeta ^2} (v \overline{v})(\lambda ) + \overline{p_1} \zeta ^2 v(\lambda )|} \\= & {} \frac{|\zeta ^2 v(\lambda ) + p_1|}{|\zeta ^2 v(\lambda ) \overline{(\zeta ^2 v(\lambda ) + p_1)}|} = \frac{|\zeta ^2 v(\lambda ) + p_1|}{|\zeta ^2 v(\lambda ) + p_1|} = 1, \end{aligned}$$

for almost all $\lambda \in \mathbb {T}$.

In [11, Theorem 1.5] it was proved that, for all $\lambda \in \mathbb {D}$,

10.5 $$\begin{aligned} \big | |\lambda |^2 s(\lambda ) - \overline{s(\lambda )}p(\lambda )\big | + |p(\lambda )|^2 + (1-|\lambda |^2)\dfrac{|s(\lambda )|^2}{4} - |\lambda |^2 = 0. \end{aligned}$$

Choose a sequence $$(r_n)_{n \ge 1}$$ such that $$ 0< r_n < 1$$ for each *n* and $$\lim \limits _{n \rightarrow \infty } r_n = 1$$. Consider $$\mu \in \mathbb {T}$$ and let $$\lambda = r_n \mu $$ in Eq. (10.5). On letting $$n \rightarrow \infty $$ we find that, for almost all $$\mu \in \mathbb {T}$$,

10.6 $$\begin{aligned} \big | |\mu |^2 s(\mu ) - \overline{s(\mu )}p(\mu )\big | + |p(\mu )|^2 + (1-|\mu |^2)\dfrac{|s(\mu )|^2}{4} - |\mu |^2 = 0. \end{aligned}$$

Note that $|\mu | = 1$ and $|p(\mu )|^2=1$, and so Eq. (10.6) is equivalent to

$$\begin{aligned} |s(\mu ) - \overline{s(\mu )}p(\mu )| = 0. \end{aligned}$$

Hence $s(\mu ) = \overline{s(\mu )}p(\mu )$ for almost all $\mu \in \mathbb {T}$. Note that for all $\lambda \in \mathbb {D}, \; h(\lambda ) = (s(\lambda ), p(\lambda )) \in \Gamma$, which means $|s(\lambda )| \le 2$ for all $\lambda \in \mathbb {D}$. Thus, for almost all $\mu \in \mathbb {T}, \; |s(\mu )| \le 2$. By [6, Proposition 3.3], $\text {deg}(h) = \text {deg}(p)$. By Lemma 10.3,

$$\begin{aligned} p(\lambda ) = \varphi _2(\lambda ) = \frac{D^{\sim 2}(\lambda )}{D(\lambda )} = \dfrac{\lambda \zeta (\lambda -\lambda _0) + \lambda p_1 {\overline{\zeta }} (1-\overline{\lambda _0}\lambda )}{{\overline{\zeta }}\{(1-\overline{\lambda _0}\lambda ) + \overline{p_1}\zeta ^2 (\lambda - \lambda _0)\}}, \end{aligned}$$

where $D(\lambda ) = {\overline{\zeta }}\{(1-\overline{\lambda _0}\lambda ) + \overline{p_1}\zeta ^2 (\lambda - \lambda _0)\}$. Since $\text {deg}(D^{\sim 2}) = 2$ and $\text {deg}(D) = 1$, then $\text {deg}(p) = 2$. Therefore, $\text {deg}(h) = 2$. $\square$

## Sharpness of the Schwarz Lemma for $\mathcal {P}$

Recall necessary conditions for a Schwarz lemma for $\mathcal {P}$ which were given in [4, Proposition 11.1].

### Proposition 11.1

[4, Proposition 11.1] Let $\lambda _0 \in \mathbb {D}\setminus \{0\}$ and $(a_0, s_0, p_0) \in \overline{\mathcal {P}}$. If $x \in Hol (\mathbb {D}, \mathcal {P})$ satisfies $x(0) = (0, 0, 0)$ and $x(\lambda _0) = (a_0, s_0, p_0)$ then $|s_0| <2$,

11.1 $$\begin{aligned} \frac{2|s_0-\overline{s}_0 p_0|+|s_0^2-4p_0|}{4-|s_0|^2} \le |\lambda _0| \end{aligned}$$

and

11.2 $$\begin{aligned} |a_0|\Big /\left| 1-\frac{\frac{1}{2}s_0{\overline{\beta }}}{1+\sqrt{1-|\beta |^2}}\right| \le |\lambda _0|, \end{aligned}$$

where $\beta = (s_0-\overline{s}_0 p_0)/(1-|p_0|^2)$ when $|p_0| < 1$ and $\beta = \frac{1}{2}s_0$ when $|p_0| = 1$.

We prove the following result.

### Theorem 11.2

Let $\lambda _0 \in \mathbb {D}\setminus \{0\}$, and $(a_0, s_0, p_0) \in \overline{\mathcal {P}}$ be such that $|s_0| <2$,

11.3 $$\begin{aligned} \dfrac{2|s_0 - p_0\overline{s}_0| + |s_{0}^{2} - 4p_0|}{4 - |s_0|^2} = |\lambda _0| \end{aligned}$$

and

11.4 $$\begin{aligned} |a_0| \; \le \; |\lambda _0| \sqrt{1-\frac{1}{4}|s_0|^2}. \end{aligned}$$

Then there exists a rational $\overline{\mathcal {P}}$-inner function $x : \mathbb {D} \rightarrow \overline{\mathcal {P}}$ such that $x(0) = (0, 0, 0)$ and $x(\lambda _0) = (a_0, s_0, p_0)$ given explicitly as follows:

(i) If $|p_0| = |\lambda _0|$, then $s_0= 0$ and $x(\lambda ) = (a(\lambda ), 0, \omega \lambda ),$ where $\omega \lambda _0 = p_0, \; \omega \in \mathbb {T}$ and
  - if $|a_0| = |\lambda _0|$, then, for $\lambda \in \mathbb {D}$, $a (\lambda ) = \kappa \lambda , \;$ where $|\kappa |=1$ and $\kappa \lambda _0 = a_0$;
  - if $$|a_0| < |\lambda _0|$$, then
    $$\begin{aligned} a(\lambda ) = \lambda \dfrac{\lambda - \lambda _0+\eta _0(1-\overline{\lambda _0}\lambda )}{1-\overline{\lambda _0}\lambda +\overline{\eta _0}(\lambda - \lambda _0)}, \; \lambda \in \mathbb {D}, \end{aligned}$$
  and $$\eta _0 = \dfrac{a_0}{\lambda _0}$$.
(ii) If $|p_0| \; <\; |\lambda _0|$, then $x(\lambda ) = (a(\lambda ), \varphi _1(\lambda ), \varphi _2(\lambda )),$ where $\varphi _1$ is defined by Eq. (10.3), $\varphi _2$ is defined by Eq. (10.4) and
  - if $|a_0| = |\lambda _0| \sqrt{1-\frac{1}{4}|s_0|^2}$, then, for $\lambda \in \mathbb {D}$,
    $$\begin{aligned} a(\lambda ) =\gamma \lambda \frac{A(\lambda )}{D(\lambda )}, \end{aligned}$$
    where $|\gamma |=1$ such that $\;\gamma \lambda _0 \sqrt{1-\frac{1}{4}|s_0|^2} = a_0$, $A(\lambda ) = b_0(1+b_1\lambda )$ is an outer polynomial of degree 1 such that
    11.5 $$\begin{aligned} |A(\lambda )|^2 = |D(\lambda )|^2-\frac{1}{4}|E(\lambda )|^2, \end{aligned}$$
    and
    11.6 $$\begin{aligned} E(\lambda ) = c \lambda ,\; \; D(\lambda ) = {\overline{\zeta }}\{(1-\overline{\lambda _0}\lambda ) + \overline{p_1}\zeta ^2 (\lambda - \lambda _0)\}, \end{aligned}$$
    with
    $$\begin{aligned} |\zeta | = 1, \; \; \; p_1 = \dfrac{p_0}{\lambda _0}, \; \; \; \; \; c = \dfrac{2}{|\lambda _0|}\{|{\overline{\lambda }}_0 - \overline{p}_0 \lambda _0 \zeta ^2| - |\lambda _0^2 \zeta ^2 - p_0|\}. \end{aligned}$$
  - if $|a_0| < |\lambda _0| \sqrt{1-\frac{1}{4}|s_0|^2}$, then, for $\lambda \in \mathbb {D}$,
    $$\begin{aligned} a(\lambda ) = \lambda \left( \frac{\lambda - \lambda _0+\mu _0(1-\overline{\lambda _0}\lambda )}{1-\overline{\lambda _0}\lambda +\overline{\mu _0}(\lambda -\lambda _0)} \right) \frac{A(\lambda )}{D(\lambda )}, \end{aligned}$$
    where $\mu _0 = \dfrac{a_0}{\lambda _0 \sqrt{1-\frac{1}{4}|s_0|^2}}$ and polynomials *A*, *E*, *D* are defined by Eqs. (11.6) and (11.5).

### Proof

Since $(a_0, s_0, p_0) \in \overline{\mathcal {P}}$, we have $(s_0, p_0) \in \Gamma$. By assumption the equality (11.3) holds. Hence, by Theorem 10.2, there exists a rational analytic function

$$\begin{aligned} \varphi : \mathbb {D} \rightarrow \Gamma : \lambda \longmapsto (s(\lambda ), p(\lambda )) \end{aligned}$$

such that $\varphi (0) = (0, 0)$ and $\varphi (\lambda _0) = (s_0, p_0)$. It is easy to see that the function $\varphi = (s, p)$ from $\mathbb {D}$ to $\Gamma$ defined as in Eq. (10.2) is a rational $\Gamma$-inner function of degree 1. By Proposition 10.4, the function $\varphi = (s, p)$ from $\mathbb {D}$ to $\Gamma$ defined by

$$\begin{aligned} s(\lambda ) = \varphi _1(\lambda ), \; p(\lambda ) = \varphi _2(\lambda ), \; \lambda \in \mathbb {D}, \end{aligned}$$

as in Eqs. (10.3) and (10.4) is a rational $\Gamma$-inner function of degree 2. By Theorem 10.1, it follows from Eq. (10.1) that $|p_0| \le |\lambda _0| \; <\; 1$. Let us consider two cases.

**Case (i)** Let $|p_0| = |\lambda _0|$. By Theorem 10.2, if $|p_0|=|\lambda _0|$, then $s_0= 0$ and the function $\varphi = (0, \omega \lambda )$ from $\mathbb {D}$ to $\Gamma$, where $\omega$ is a complex number of unit modulus such that $\omega \lambda _0 = p_0$, is a rational $\Gamma$-inner function of degree 1. Since $s_0=0$, the assumption (11.4) $|a_0| \le |\lambda _0| \sqrt{1-\frac{1}{4}|s_0|^2}$ is equivalent to $|a_0| \le |\lambda _0|$. It is easy to see that, for $\lambda \in \mathbb {D}$, $s(\lambda ) = \dfrac{E(\lambda )}{D(\lambda )}$ and $p(\lambda ) = \dfrac{D^{\sim n}}{D}(\lambda )$, where polynomials $E(\lambda )=0$ and $D(\lambda ) = \overline{\omega _1}$ and $\omega _1^2=\omega$. By Theorem 7.8, we can construct a rational $\overline{\mathcal {P}}$-inner function

$$\begin{aligned} x = \left( cB \dfrac{A}{D}, 0, \omega \lambda \right) , \text { for an arbitrary finite Blaschke product } B \text { and } |c| = 1, \end{aligned}$$

where *A* is a non-zero constant such that

$$\begin{aligned} |A(\lambda )|^2 = |D(\lambda )|^2-\frac{1}{4}|E(\lambda )|^2= |D(\lambda )|^2= | \overline{\omega _1}|^2 =1. \end{aligned}$$

It is sufficient to construct $a : \mathbb {D} \rightarrow \mathbb {C}$ of the form $a = c B$ with $|c|=1$ such that $a(0) = 0$ and $a(\lambda _0) = a_0$.

If $|a_0| = |\lambda _0|$, then, for $\lambda \in \mathbb {D}$, we define $a(\lambda ) = \kappa \lambda \; \; \text {for} \;\;\lambda \in \mathbb {D},$ where $|\kappa |=1$ and $\kappa \lambda _0 = a_0$. It is easy to see that $a(0) = 0$ and $a(\lambda _0) = a_0$.

Let $|a_0| \; <\; |\lambda _0|$, and let $\eta _0 {\mathop {=}\limits ^{def }} \frac{a_0}{\lambda _0},$ it is clear that $\eta _0 \in \mathbb {D}$. Let us define *a* by the formula

$$\begin{aligned} a(\lambda ) = \lambda B_{\eta _0}^{-1} \circ B_{\lambda _0}(\lambda ) \; \; \text {for} \;\;\lambda \in \mathbb {D}. \end{aligned}$$

Here the Blaschke factors are defined by

$$\begin{aligned} B_{\eta _0}^{-1}(z) = \frac{z+\eta _0}{1+\overline{\eta _0}z} \text { and } B_{\lambda _0}(z) = \frac{z-\lambda _0}{1-\overline{\lambda _0}z} \; \text {for} \; z \in \mathbb {D}. \end{aligned}$$

It is easy to see that $a(0) = 0$ and $a(\lambda _0) = a_0$.

Define a rational $\overline{\mathcal {P}}$-inner function $x : \mathbb {D} \rightarrow \overline{\mathcal {P}}$ by $x(\lambda ) = (a(\lambda ), 0, \omega \lambda ),$ where $\omega \lambda _0 = p_0, \; \omega \in \mathbb {T}$ and

- if $|a_0| = |\lambda _0|$, then, for $\lambda \in \mathbb {D}$, $a (\lambda ) = \kappa \lambda , \;$ where $|\kappa |=1$ and $\kappa \lambda _0 = a_0$;
- if $$|a_0| < |\lambda _0|$$, then
  $$\begin{aligned} a(\lambda ) = \lambda \dfrac{\lambda - \lambda _0+\eta _0(1-\overline{\lambda _0}\lambda )}{1-\overline{\lambda _0}\lambda +\overline{\eta _0}(\lambda - \lambda _0)}, \; \lambda \in \mathbb {D}, \end{aligned}$$
  and $\eta _0 = \dfrac{a_0}{\lambda _0}$. This function *x* satisfies the conditions $x(0) = (0, 0, 0)$ and $x(\lambda _0) = (a_0, s_0, p_0)$.

**Case (ii)** Let $|p_0| \; <\; |\lambda _0|$. By Lemma 10.3, for the rational $\Gamma$-inner function $\varphi = (s, p)$ from $\mathbb {D}$ to $\Gamma$ defined by

$$\begin{aligned} s(\lambda ) = \varphi _1(\lambda ), \; p(\lambda ) = \varphi _2(\lambda ), \; \lambda \in \mathbb {D}, \end{aligned}$$

as in Eqs. (10.3) and (10.4), there exist polynomials *E* and *D*

11.7 $$\begin{aligned} E(\lambda ) = c \lambda ,\; \; D(\lambda ) = {\overline{\zeta }}\{(1-\overline{\lambda _0}\lambda ) + \overline{p_1}\zeta ^2 (\lambda - \lambda _0)\}, \end{aligned}$$

where

$$\begin{aligned} |\zeta | = 1, \; \; \; p_1 = \dfrac{p_0}{\lambda _0}, \; \; \; \; \; c = \dfrac{2}{|\lambda _0|}\{|{\overline{\lambda }}_0 - \overline{p}_0 \lambda _0 \zeta ^2| - |\lambda _0^2 \zeta ^2 - p_0|\}, \end{aligned}$$

such that $s = \dfrac{E}{D}$ and $p= \dfrac{D^{\sim 2}}{D}$. Moreover, $E^{\sim 2} = E$ and $|E(\lambda )| \le 2 |D(\lambda )|$ on $\overline{\mathbb {D}}$.

Then, by Theorem 7.8, we can construct a rational $\overline{\mathcal {P}}$-inner function

$$\begin{aligned} x = \left( cB \dfrac{A}{D}, \dfrac{E}{D}, \dfrac{D^{\sim n}}{D}\right) , \text { for an arbitrary finite Blaschke product } B \text { and } |c| = 1, \end{aligned}$$

where $A= b_0(1+b_1 \lambda )$ is an outer polynomial of degree 1 such that

11.8 $$\begin{aligned} |A(\lambda )|^2 = |D(\lambda )|^2-\frac{1}{4}|E(\lambda )|^2. \end{aligned}$$

We would like to construct $a : \mathbb {D} \rightarrow \mathbb {C}$ of the form $a = c B \dfrac{A}{D}$ such that $a(0) = 0$ and $a(\lambda _0) = a_0$.

Since, for $$\lambda \in \mathbb {D}$$,

$$\begin{aligned} \frac{|A(\lambda )|^2}{|D(\lambda )|^2} = 1-\frac{1}{4}|s(\lambda )|^2, \end{aligned}$$

we get $\big |\dfrac{A}{D}(\lambda _0) \big | = \sqrt{1-\frac{1}{4}|s_0|^2}$. Let $B(\lambda ) = \lambda \tilde{B}(\lambda ),$ with some finite Blaschke product $\tilde{B}$. Then $B(0) = 0$, and so $a(0) = 0$. Recall we require $a(\lambda _0) = a_0$,

$$\begin{aligned} a_0= & {} a(\lambda _0) = c B(\lambda _0) \dfrac{A}{D}(\lambda _0)\\= & {} c \lambda _0 \tilde{B}(\lambda _0) \sqrt{1-\frac{1}{4}|s_0|^2}, \end{aligned}$$

for some $|c| = 1$. By assumption (11.4),

$$\begin{aligned} |a_0| \; \le \; |\lambda _0| \sqrt{1-\frac{1}{4}|s_0|^2} \;\text {and} \; \; |s_0| <2, \,\; \text { thus } \frac{|a_0|}{|\lambda _0| \sqrt{1-\frac{1}{4}|s_0|^2}} \; \le \; 1. \end{aligned}$$

Suppose $|a_0| < |\lambda _0| \sqrt{1-\frac{1}{4}|s_0|^2}$, and let

$$\begin{aligned} \mu _0 {\mathop {=}\limits ^{def }} \frac{a_0}{\lambda _0 \sqrt{1-\frac{1}{4}|s_0|^2}}. \end{aligned}$$

It is clear that $\mu _0 \in \mathbb {D}$. We need to find $\tilde{B} : \mathbb {D} \rightarrow \mathbb {D}$ such that $\tilde{B}(\lambda _0) = \mu _0$. Let $\tilde{B}= B_{\mu _0}^{-1} \circ B_{\lambda _0}$, where

$$\begin{aligned} B_{\mu _0}^{-1}(z) = \frac{z+\mu _0}{1+\overline{\mu _0}z} \text { and } B_{\lambda _0}(z) = \frac{z-\lambda _0}{1-\overline{\lambda _0}z}. \end{aligned}$$

Then, for all $z \in \mathbb {D}$,

$$\begin{aligned} \tilde{B}(z)= & {} B_{\mu _0}^{-1} \circ B_{\lambda _0}(z) = \frac{\dfrac{z-\lambda _0}{1-\overline{\lambda _0}z}+\mu _0}{1+\overline{\mu _0}\left( \dfrac{z-\lambda _0}{1-\overline{\lambda _0}z}\right) }\\= & {} \frac{z-\lambda _0+\mu _0(1-\overline{\lambda _0}z)}{1-\overline{\lambda _0}z+\overline{\mu _0}(z-\lambda _0)}. \end{aligned}$$

Let us define $a : \mathbb {D} \rightarrow \mathbb {C}$, for $\lambda \in \mathbb {D}$, by

$$\begin{aligned} a(\lambda ) = \lambda \tilde{B}(\lambda ) \dfrac{A(\lambda )}{D(\lambda )}= \lambda \left( \frac{\lambda - \lambda _0+\mu _0(1-\overline{\lambda _0}\lambda )}{1-\overline{\lambda _0}\lambda +\overline{\mu _0}(\lambda -\lambda _0)} \right) \frac{A(\lambda )}{D(\lambda )}. \end{aligned}$$

Note that $a(0) = 0$ and $\tilde{B}(\lambda _0) = \dfrac{\mu _0(1-\overline{\lambda _0}\lambda _0)}{1-\overline{\lambda _0}\lambda _0} = \mu _0$. Therefore,

$$\begin{aligned} a(\lambda _0)= & {} \lambda _0 \mu _0 \frac{A(\lambda _0)}{D(\lambda _0)} \\= & {} \lambda _0 \frac{a_0}{\lambda _0 \sqrt{1-\frac{1}{4}|s_0|^2}} \sqrt{1-\frac{1}{4}|s_0|^2} \\= & {} a_0. \end{aligned}$$

Hence, in the case when $|p_0| \; <\; |\lambda _0|$, we define a rational $\overline{\mathcal {P}}$-inner function $x : \mathbb {D} \rightarrow \overline{\mathcal {P}}$ by $x(\lambda ) = (a(\lambda ), \varphi _1(\lambda ), \varphi _2(\lambda )), \;\ \text {for all } \;\; \lambda \in \mathbb {D},$ where $\varphi _1$ is defined by Eq. (10.3), $\varphi _2$ is defined by Eq. (10.4) and

- if $|a_0| = |\lambda _0| \sqrt{1-\frac{1}{4}|s_0|^2}$, then, for $\lambda \in \mathbb {D}$,
  $$\begin{aligned} a(\lambda ) = \gamma \lambda \frac{A(\lambda )}{D(\lambda )}, \end{aligned}$$
  where $|\gamma |=1$ such that $\;\gamma \lambda _0 \sqrt{1-\frac{1}{4}|s_0|^2} = a_0$.
- if $$|a_0| < |\lambda _0| \sqrt{1-\frac{1}{4}|s_0|^2}$$, then
  $$\begin{aligned} a(\lambda ) = \lambda \left( \frac{\lambda - \lambda _0+\mu _0(1-\overline{\lambda _0}\lambda )}{1-\overline{\lambda _0}\lambda +\overline{\mu _0}(\lambda -\lambda _0)} \right) \frac{A(\lambda )}{D(\lambda )}, \end{aligned}$$
  where $\mu _0 = \dfrac{a_0}{\lambda _0 \sqrt{1-\frac{1}{4}|s_0|^2}}$, and polynomials *A*, *E* and *D* are defined by Eqs. (11.7) and (11.8).

One can verify that a suitable choice of *A* is $A(\lambda ) = b_0(1+b_1\lambda )$, where

$$\begin{aligned} |b_0|^2 = |1-\overline{p_1}\zeta ^2 \lambda _0|^2 \;\; \text {and} \;\; |b_1|^2 = 2 \; \frac{|\lambda _0 \zeta ^2 - p_1|}{|1-\overline{p_1}\zeta ^2 \lambda _0|} - 1. \end{aligned}$$

$$\square $$

### Theorem 11.3

Let $\lambda _0 \in \mathbb {D}\setminus \{0\}$ and $(a_0, s_0, p_0) \in \overline{\mathcal {P}}$. Then the following conditions are equivalent:

(i) there exists a rational $\overline{\mathcal {P}}$-inner function $x=(a, s, p)$, $x : \mathbb {D} \rightarrow \overline{\mathcal {P}}$ such that $x(0) = (0, 0, 0)$ and $x(\lambda _0) = (a_0, s_0, p_0)$;
(ii) there exists an analytic function $x=(a, s, p)$, $x : \mathbb {D} \rightarrow \overline{\mathcal {P}}$ such that $x(0) = (0, 0, 0)$ and $x(\lambda _0) =(a_0, s_0, p_0)$, and $|a_0| \; \le \; |\lambda _0| \sqrt{1-\frac{1}{4}|s_0|^2}$;
(iii) 11.9 $$\begin{aligned} \dfrac{2|s_0 - p_0\overline{s}_0| + |s_{0}^{2} - 4p_0|}{4 - |s_0|^2} \le |\lambda _0|, \;\;|s_0| <2, \end{aligned}$$
  and
  11.10 $$\begin{aligned} |a_0| \; \le \; |\lambda _0| \sqrt{1-\frac{1}{4}|s_0|^2}. \end{aligned}$$

### Proof

We shall prove below that (i) $\Longleftrightarrow$ (iii), from which it will follow trivially that (i) $\Rightarrow$ (ii).

(ii) $\Rightarrow$ (i) Suppose (ii) holds, that is, there exists an analytic function $x_1=(a', s', p')$, $x_1 : \mathbb {D} \rightarrow \overline{\mathcal {P}}$ such that $x_1(0) = (0, 0, 0)$ and $x_1(\lambda _0) = (a_0, s_0, p_0)$. By Lemma 6.2, $h_1=(s',p'): \mathbb {D} \rightarrow \Gamma$ is an analytic function such that $h_1(0) = (0, 0)$ and $h_1(\lambda _0) = (s_0, p_0)$.

By [18, Theorem 4] (see also [3, Theorem 8.1]), there exists a rational $\Gamma$-inner function $h: \mathbb {D} \rightarrow \Gamma$ satisfying $h(0) = (0, 0)$ and $h(\lambda _0) = (s_0, p_0)$. Let *E* and *D* be polynomials as in Eq. (7.1) (see [6, Proposition 2.2]) with $h= \left( \dfrac{E}{D}, \dfrac{D^{\sim n}}{D}\right)$ on $\mathbb {D}$, where $n = \deg h$. By Theorem 7.8, we can construct a rational $\overline{\mathcal {P}}$-inner function

$$\begin{aligned} x = \left( a,\dfrac{E}{D}, \dfrac{D^{\sim n}}{D}\right) =\left( cB \dfrac{A}{D},\dfrac{E}{D}, \dfrac{D^{\sim n}}{D}\right) , \end{aligned}$$

for any finite Blaschke product *B* and $$c \in \mathbb {C}$$ with $$ |c| = 1,$$ where *A* is an outer polynomial such that

$$\begin{aligned} |A(\lambda )|^2 = |D(\lambda )|^2-\frac{1}{4}|E(\lambda )|^2, \;\; \text {for} \;\;\lambda \in \mathbb {T}. \end{aligned}$$

Hence, for $$\lambda \in \mathbb {D}$$,

$$\begin{aligned} \frac{|A(\lambda )|^2}{|D(\lambda )|^2} = 1-\frac{1}{4}|s(\lambda )|^2. \end{aligned}$$

Thus we get $\big |\dfrac{A}{D}(\lambda _0) \big | = \sqrt{1-\frac{1}{4}|s_0|^2}$. By assumption, $|a_0| \le |\lambda _0| \sqrt{1-\frac{1}{4}|s_0|^2}$. As in Theorem 11.2, to satisfy conditions $x(0) = (0, 0, 0)$ and $x(\lambda _0) = (a_0, s_0, p_0)$, we define a function *a* the following way:

- if $|a_0| = |\lambda _0| \sqrt{1-\frac{1}{4}|s_0|^2}$, then, for $\lambda \in \mathbb {D}$,
  $$\begin{aligned} a(\lambda ) = \gamma \lambda \frac{A(\lambda )}{D(\lambda )}, \end{aligned}$$
  where $|\gamma |=1$ is such that $\;\gamma \lambda _0 \sqrt{1-\frac{1}{4}|s_0|^2} = a_0$.
- if $$|a_0| < |\lambda _0| \sqrt{1-\frac{1}{4}|s_0|^2}$$, then
  $$\begin{aligned} a(\lambda ) = \lambda \left( \frac{\lambda - \lambda _0+\mu _0(1-\overline{\lambda _0}\lambda )}{1-\overline{\lambda _0}\lambda +\overline{\mu _0}(\lambda -\lambda _0)} \right) \frac{A(\lambda )}{D(\lambda )}, \end{aligned}$$
  where $\mu _0 = \dfrac{a_0}{\lambda _0 \sqrt{1-\frac{1}{4}|s_0|^2}}$. Therefore, condition (i) holds.

(iii) $\Rightarrow$ (i) By Theorem 10.1, since condition (iii) holds, there exists an analytic function $h_1: \mathbb {D} \rightarrow \Gamma$ such that $h_1(0) = (0, 0)$ and $h_1(\lambda _0) = ( s_0, p_0)$. By [18, Theorem 4] (see also [3, Theorem 8.1]), there exists a rational $\Gamma$-inner function $h=(s, p): \mathbb {D} \rightarrow \Gamma$ satisfying $h(0) = (0, 0)$ and $h(\lambda _0) = (s_0, p_0)$. Let *E* and *D* be polynomials as in Eq. (7.1) (see [6, Proposition 2.2]) with $h= \left( \dfrac{E}{D}, \dfrac{D^{\sim n}}{D}\right)$ on $\mathbb {D}$, where $n = \deg h$. By Theorem 7.8, we can construct a rational $\overline{\mathcal {P}}$-inner function

$$\begin{aligned} x = \left( a,\dfrac{E}{D}, \dfrac{D^{\sim n}}{D}\right) =\left( cB \dfrac{A}{D},\dfrac{E}{D}, \dfrac{D^{\sim n}}{D}\right) , \end{aligned}$$

for any finite Blaschke product *B* and $$c \in \mathbb {C}$$ with $$ |c| = 1,$$ where *A* is an outer polynomial such that

$$\begin{aligned} |A(\lambda )|^2 = |D(\lambda )|^2-\frac{1}{4}|E(\lambda )|^2. \end{aligned}$$

To satisfy the conditions $x(0) = (0, 0, 0)$ and $x(\lambda _0) = (a_0, s_0, p_0)$, we define a function *a* as in Part (ii) $\Rightarrow$ (i).

Note that in the case when

$$\begin{aligned} \dfrac{2|s_0 - p_0\overline{s}_0| + |s_{0}^{2} - 4p_0|}{4 - |s_0|^2} = |\lambda _0|,\;\;|s_0| <2, \end{aligned}$$

and

$$\begin{aligned} |a_0| \; \le \; |\lambda _0| \sqrt{1-\frac{1}{4}|s_0|^2} \end{aligned}$$

Theorem 11.2 gives the construction of an interpolating rational $\overline{\mathcal {P}}$-inner function $x=(a, s, p)$, $x : \mathbb {D} \rightarrow \overline{\mathcal {P}}$ such that $x(0) = (0, 0, 0)$ and $x(\lambda _0) = (a_0, s_0, p_0)$.

(i) $\Rightarrow$ (iii) Suppose (i) holds, that is, there exists a rational $\overline{\mathcal {P}}$-inner function $x=(a, s, p)$, $x : \mathbb {D} \rightarrow \overline{\mathcal {P}}$ such that $x(0) = (0, 0, 0)$ and $x(\lambda _0) = (a_0, s_0, p_0)$. Let $\deg x = (m, n)$.

By Lemma 6.2, $h=(s,p)$ is a rational $\Gamma$-inner function of degree *n*. Note that $h(0) = (0,0)$ and $h(\lambda _0) = (s_0, p_0)$. By Theorem 10.1, $|s_0| \; <\; 2$ and

$$\begin{aligned} \dfrac{2|s_0 - p_0\overline{s_0}| + |s_{0}^{2} - 4p_0|}{4 - |s_0|^2} \le |\lambda _0|. \end{aligned}$$

By Theorem 7.5, there exist polynomials *A*, *E*, *D* such that $E^{\sim n} = E$, $D(\lambda ) \ne 0$ on $\overline{\mathbb {D}}$, *A* is an outer polynomial such that $|A(\lambda )|^2 = |D(\lambda )|^2 - \frac{1}{4}|E(\lambda )|^2$ on $\mathbb {T}$, $|E(\lambda )| \le 2|D(\lambda )|$ on $\overline{\mathbb {D}}$ and

$$\begin{aligned} x=\left( c B \dfrac{A}{D}, \dfrac{E}{D}, \dfrac{D^{\sim n}}{D}\right) \;\; \text {on} \;\;\overline{\mathbb {D}} \end{aligned}$$

for some finite Blaschke product *B* and $|c|=1$. The function

$$\begin{aligned} \lambda \mapsto a(\lambda ) = c B (\lambda )\dfrac{A}{D}(\lambda ) \end{aligned}$$

is an analytic map from $\mathbb {D}$ to $\overline{\mathbb {D}}$ such that $a(0) = 0$ and $a(\lambda _0) = a_0$. Note that *A* and *D* are outer polynomials on $\overline{\mathbb {D}}$, and so

$$\begin{aligned} f(\lambda ) =\frac{a(\lambda )}{\left( \dfrac{A}{D}(\lambda ) \right) }= c B (\lambda ) \end{aligned}$$

is an analytic map from $\mathbb {D}$ to $\overline{\mathbb {D}}$ such that $f(0) = 0$. By the classical Schwarz lemma we have

$$\begin{aligned} |f(\lambda )| = \left| \frac{a(\lambda )}{\dfrac{A}{D}(\lambda )} \right| \le | \lambda | \;\; \text {for}\;\;\lambda \in \mathbb {D}. \end{aligned}$$

Since

$$\begin{aligned} \big |\dfrac{A}{D}(\lambda ) \big |^2{} & {} = 1- |s (\lambda )|^2\;\; \text {for} \;\; \lambda \in \mathbb {D},\\ |f(\lambda )|{} & {} = \left| \frac{a(\lambda )}{\sqrt{1-\frac{1}{4}|s (\lambda )|^2}} \right| \;\; \text {for} \;\; \lambda \in \mathbb {D}. \end{aligned}$$

Thus

$$\begin{aligned} |a_0| = |a(\lambda _0)| =|f(\lambda _0)| \sqrt{1-\frac{1}{4}|s (\lambda _0)|^2} \le | \lambda _0| \sqrt{1-\frac{1}{4}|s_0|^2}. \end{aligned}$$

Therefore, condition (iii) holds. $\square$
